# Advancing diagnostic equity through artificial intelligence chest radiograph screening for osteoporosis in Asian populations

**DOI:** 10.1038/s41746-026-02484-x

**Published:** 2026-03-19

**Authors:** Shu-Han Chen, Ray-E Chang, Chia-En Lien, Dun-Jhu Yang, Pei Yao, Meng-Lu Wu, Kun-Hui Chen

**Affiliations:** 1 Department of Family Medicine, St. Paul’s Hospital, Taoyuan, Taiwan; 2 Health Management Center, St. Paul’s Hospital, Taoyuan, Taiwan; 3 Institute of Health Policy and Management, College of Public Health, National Taiwan University, Taipei, Taiwan; 4 Acer Medical Inc., New Taipei City, Taiwan; 5 Acer Inc., Taipei City, Taiwan; 6 Information Technology Department, St. Paul’s Hospital, Taoyuan, Taiwan; 7 Department of Orthopedic Surgery, Taichung Veterans General Hospital, Taichung, Taiwan; 8 Department of Post-Baccalaureate Medicine, College of Medicine, National Chung Hsing University, Taichung, Taiwan; 9 Department of Computer Science and Information Engineering, Providence University, Taichung, Taiwan

**Keywords:** Diseases, Health care, Medical research

## Abstract

Early identification of abnormal bone mineral density (BMD) through opportunistic screening is critical for preventing osteoporotic fractures. We validated an AI model in 2384 asymptomatic adults (57.7% female; mean age 43.6 years) undergoing health examinations in Taiwan. Using DXA as the reference, the model identified 255 suspected abnormal BMD cases, with 94 (3.9%) DXA-confirmed positive. Population-level performance was robust, yielding an AUC of 0.95 (95% CI 0.93–0.99) and sensitivity of 79.7% (95% CI 71.3–86.5%). Although BMI distributions paralleled East Asian regional trends, intersectional subgroup analyses remain exploratory due to small event counts. Decision curve analysis indicated superior net benefit for AI-based referral over “refer all” or “refer none” strategies, particularly for women with normal BMI (18.5–23 kg/m²). This AI tool offers precise triage for Asian health examination populations, though further validation in multi-center cohorts is required to confirm broad generalizability.

## Introduction

Osteoporosis is a major yet under-recognized public health concern, affecting over 200 million individuals globally and contributing to more than 8.9 million fractures annually, with one in three women and one in five men over age 50 expected to sustain a fragility fracture during their lifetime^1,2^. As populations age—especially in Asia where demographic transitions are accelerating—the incidence of osteoporosis-related fractures is projected to rise dramatically, with over 50% of global hip fractures predicted to occur in Asia by 2050^3,4^. Despite this looming burden, current national screening guidelines remain narrowly focused on postmenopausal women aged ≥65 or younger women with specific risk factors, and they provide no general recommendation for men^5–7^. These constraints result in limited routine screening coverage for several potentially at-risk groups, including younger adults, men, and individuals with non-classical indicators such as normal or high body mass index (BMI)^8–10^.

Even among eligible groups, the clinical uptake of dual-energy X-ray absorptiometry (DXA)—the gold standard for osteoporosis diagnosis—remains strikingly low. In both resource-limited and well-resourced countries, fewer than 20% of older women and under 5% of men undergo DXA screening in practice^6,11,12^. Furthermore, more than half of all fragility fractures occur in individuals with osteopenic or even normal bone mineral density (BMD), underscoring the limited sensitivity of T-score thresholds alone^13^ and the existence of a substantial “osteoporosis care gap”.

Despite this burden, most national screening guidelines restrict DXA testing to postmenopausal women aged ≥65 years or younger women with specific clinical risk factors (Supplementary Table 1). The 2014 National Osteoporosis Foundation (NOF)^14^ Clinician’s Guide and the 2023 International Society for Clinical Densitometry (ISCD)^15^ positions both define osteoporosis based on DXA-derived T- or Z-scores but restrict testing to individuals meeting clinical indications, while the 2025 U.S. Preventive Services Task Force (USPSTF)^16^ statement continues to conclude that evidence is insufficient to recommend screening in men. As a result, younger adults and men are less consistently captured by guideline-based screening strategies, underscoring the need for more equitable and scalable approaches.

Additionally, recent meta-analytic evidence has revealed substantial diagnostic discordance between DXA and quantitative computed tomography (QCT), with QCT identifying significantly more osteoporosis cases—particularly among men and older adults—suggesting that DXA-based definitions may underestimate the true disease burden^17^.

Artificial intelligence (AI) has recently emerged as a promising tool to extend fracture risk detection using routinely acquired medical images. Most existing AI approaches for opportunistic osteoporosis screening have been developed using computed tomography (CT) images rather than chest radiographs, which limits scalability in routine preventive settings^18^. Chest radiographs (CXR), performed more than 800 million times annually worldwide, often incidentally visualize thoracic vertebrae and thus represent an untapped resource for bone health assessment^19^. Deep learning models, especially convolutional neural networks (CNNs), have been trained to extract bone-related features from CXRs to estimate BMD or predict DXA-defined osteoporosis^20,21^. Prior studies reported high diagnostic accuracy (area under the curve [AUC] >0.88) for these AI models^22–24^, and a randomized controlled trial has shown that AI-assisted CXR triage significantly boosts downstream DXA referrals and new osteoporosis diagnoses^20^. Nevertheless, most existing models were trained on retrospective or homogeneous datasets with limited generalizability across real-world populations^11^^,^^25^^,^^26^.

To address these limitations, we applied an updated version of a previously validated deep learning model for osteoporosis detection using chest radiographs^27^ to a large retrospective cohort from a preventive health management center in northern Taiwan. Unlike prior studies in elderly or clinical populations, this evaluation used a relatively healthy, asymptomatic, self-selected screening Asian cohort reflective of community demographics. We conducted exploratory subgroup analyses across 12 strata defined by sex, age (<50 vs ≥50 years), and World Health Organization (WHO) Asia-Pacific BMI classification (<18.5, 18.5–23, ≥23.0 kg/m²)^28^ to characterize performance patterns. These analyses offer hypothesis-generating insights into AI-assisted screening potential.

This study aimed to examine the feasibility and performance of AI-enabled chest radiograph (AI-CXR) screening within the context of routine preventive care. Specifically, we sought to evaluate the capability of an AI-CXR tool to flag suspected abnormal bone mineral density (saBMD), with the goal of identifying individuals who meet clinical criteria for further diagnostic confirmation. In this study, abnormal BMD was defined as osteoporosis (T-score ≤ −2.5) for participants aged ≥50 years, following the WHO 1994 threshold, and as low bone mineral density for age (Z-score ≤ −2.0) for those aged <50 years, according to the ISCD Official Positions. By establishing these clear, age-stratified thresholds, we aimed to provide a robust assessment of AI-CXR as a potential opportunistic screening tool.

## Results

### Study population characteristics

A total of 2384 participants were included in the final analysis, comprising 1376 females (57.7%) and 1008 males (42.3%), with a mean age of 43.6 years (SD 9.9; range 20–99). The mean body mass index (BMI) was 23.4 ± 3.9 kg/m². According to World Health Organization (WHO) criteria for Asian populations, 6.2% of participants were underweight (BMI < 18.5 kg/m²), 45.5% had normal weight (18.5–23 kg/m²), and 46.3% were overweight (BMI ≥ 23 kg/m²). The mean lumbar spine bone mineral density (BMD) was 1.13 ± 0.17 g/cm², with a mean T-score of −0.33 ± 1.24. AI-flagged suspected abnormal BMD (saBMD) was identified in 255 participants (10.7%), while DXA-confirmed abnormal BMD was present in 94 participants (3.9%). The majority of chest radiographs (CXRs) were evaluable (98.0%).

Sex-specific comparisons showed that males had a higher mean BMI than females (24.9 vs. 22.3 kg/m²; *p* < 0.0001). Males were more frequently classified as overweight (BMI ≥ 23 kg/m²; 68.5% vs. 30.1%; *p* < 0.0001), whereas underweight status (BMI < 18.5 kg/m²) was less common in males than in females (2.4% vs. 8.9%; *p* < 0.0001). Mean BMD and T-scores were similar between sexes; however, the prevalence of saBMD was significantly lower in males than in females (8.3% vs. 12.3%, *p* = 0.0002). A higher proportion of unevaluable CXRs was observed in males compared with females (4.3% vs. 0.4%; *p* < 0.0001) (Table 1).

**Table 1:** Baseline characteristics by sex

Variable	Overall (*n* = 2384)	Male (*n* = 1008)	Female (*n* = 1376)	*p*-value
Age, Mean ± SD (years)	43.6 ± 9.9 (20–99)	42.6 ± 9.5	44.4 ± 10.1	<0.0001
Age ≥50, *n* (%)	662 (28%)	247 (24.5%)	415 (30.1%)	0.002
Age <50, *n* (%)	1722 (72%)	761 (75.5%)	961 (69.9%)	—
BMI, Mean ± SD (kg/m²)	23.4 ± 3.9	24.89 ± 3.68	22.32 ± 3.60	<0.0001
BMI < 18.5, *n* (%)	147 (6.2%)	24 (2.4%)	123 (8.9%)	<0.0001
23 > BMI > 18.5, *n* (%)	1084 (45.5%)	294 (29.2%)	790 (57.4%)	<0.0001
BMI ≥ 23, *n* (%)	1104 (46.3%)	690 (68.5%)	414 (30.1%)	<0.0001
Bone mass density				
BMD, Mean ± SD (g/cm²)	1.13 ± 0.17	1.14 ± 0.14	1.13 ± 0.16	0.1124
T-score, Mean ± SD	−0.33 ± 1.24	−0.31 ± 1.13	−0.40 ± 1.29	0.09
Z-score, mean ± SD	0.44 ± 1.13	0.34 ± 1.11	0.50 ± 1.11	0.0005
saBMD (Suspected abnormal BMD), *n* (%)	255 (10.7%)	80 (8.3%)	168 (12.3%)	0.0002
Non-saBMD, *n* (%)	2129 (89%)	885 (91.7%)	1202 (87.7%)	<0.0001
Collected images				
Evaluable CXRs, *n* (%)	2335 (98%)	965 (95.7%)	1370 (99.6%)	<0.0001
Not Evaluable CXRs, *n* (%)	49 (2%)	43 (4.3%)	6 (0.4%)	<0.0001

Values are presented as mean ± standard deviation for continuous variables and as counts (%) for categorical variables. Statistical testing: Student’s *t*-test for continuous variables and χ^2^ test for categorical variables. BMI categories follow WHO Asia-Pacific definitions (<18.5, 18.5–23, ≥23 kg/m²). saBMD = suspected abnormal bone mineral density (T-score ≤ −2.5 for participants ≥50 years, or Z-score ≤ −2.0 for participants <50 years). Evaluable CXR indicates images meeting predefined quality-control criteria; non-evaluable CXR indicates images excluded for insufficient image quality.

Participants aged ≥50 years (*n* = 662, 28%) had lower mean BMD (1.08 vs. 1.16 g/cm²; *p* < 0.0001) and lower mean T-scores (−0.80 vs. −0.15; *p* < 0.0001) than those aged <50 years, along with a higher prevalence of saBMD (19.0% vs. 7.5%; *p* < 0.0001). Older participants also had a higher mean BMI (24.3 vs. 23.0 kg/m²; *p* < 0.0001) and a higher prevalence of overweight status (BMI ≥ 23 kg/m², 55.4% vs. 47.2%) (Supplementary Table 2). Across BMI categories, underweight participants (BMI < 18.5 kg/m²) had the lowest mean BMD (1.07 g/cm²) and T-scores (−0.90), and the highest prevalence of saBMD (22.4%), whereas overweight participants (BMI ≥ 23 kg/m²) had the highest mean BMD (1.16 g/cm²), highest T-scores (−0.14), and the lowest saBMD prevalence (6.2%). Normal-weight participants (18.5–23 kg/m²) showed intermediate values across these measures (Supplementary Table 3).

Baseline characteristics were further examined across 12 demographic subgroups defined by sex, age (<50 or ≥50 years), and BMI category (<18.5, 18.5–23, ≥23 kg/m²). Supplementary Table 4 provides a detailed breakdown of these characteristics, illustrating the demographic heterogeneity and clinical profiles of each intersectional stratum in our cohort. The prevalence of saBMD varied across subgroups, ranging from 3.1% in females aged <50 years with BMI ≥ 23 kg/m² to 41.7% in females aged ≥50 years with BMI < 18.5 kg/m². Among males aged <50 years with BMI < 18.5 kg/m², the prevalence of saBMD was 31.6%.

**Fig. 1:**
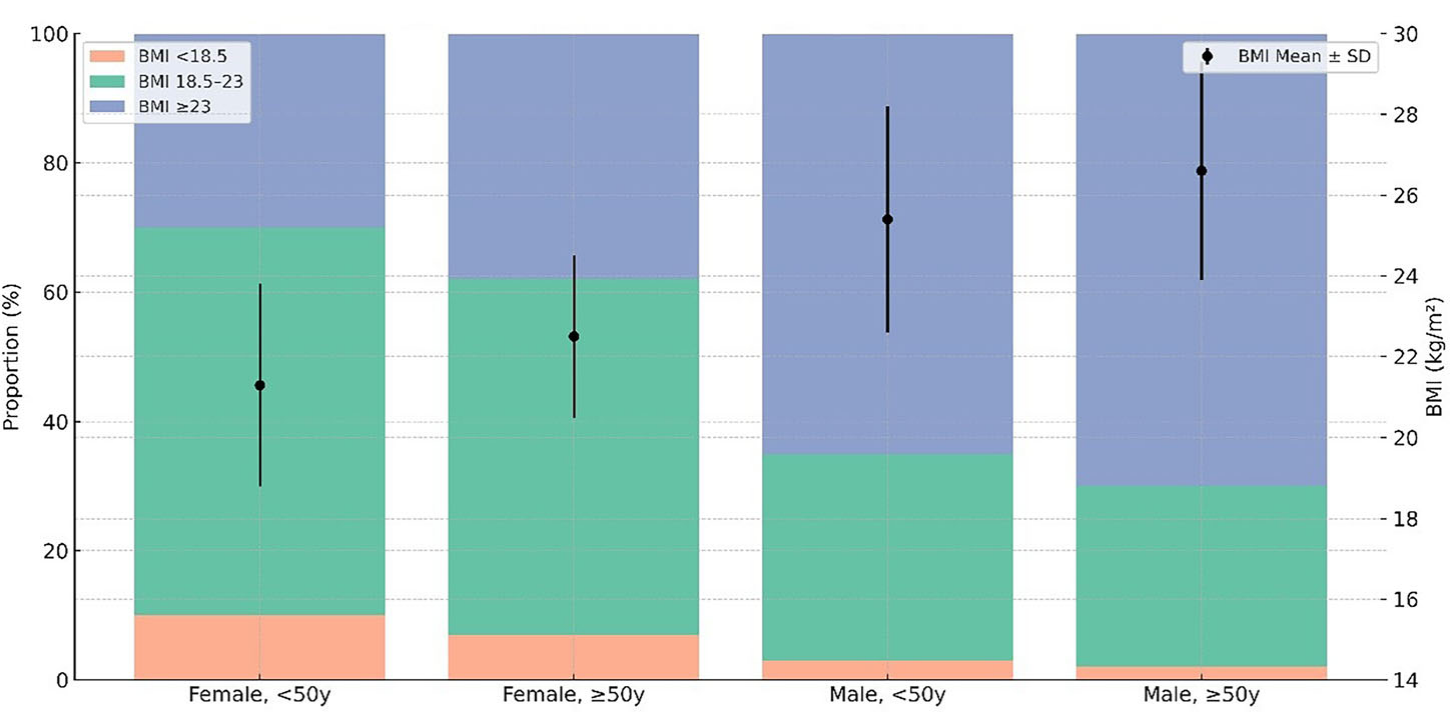
Distribution of body mass index (BMI) categories by sex and age group, with mean BMI values. Stacked bar plots depict the proportion of participants classified as BMI < 18.5, 18.5–23, and ≥23 kg/m² across four sex–age strata. Black points indicate subgroup mean BMI, with vertical bars representing ±1 standard deviation. Differences in BMI distribution and mean BMI were observed across sex and age groups, with higher mean BMI and a greater proportion of BMI ≥ 23 kg/m² in men, particularly in those aged ≥50 years, and a higher proportion of BMI < 18.5 kg/m² in women aged <50 years. These descriptive data provide contextual information on cohort characteristics relevant to subsequent subgroup analyses.

As shown in Fig. 1, Men aged ≥50 years had the highest mean BMI (26.7 kg/m²) and the greatest inter-individual variability, whereas women aged <50 years had the lowest mean BMI (21.3 kg/m²) and the highest proportion of underweight individuals. Individuals with normal BMI (18.5–23 kg/m²) constituted the largest subgroup across both sexes and age strata. Comparisons with East Asian national health surveys indicated that our study cohort’s BMI distribution parallels regional trends, particularly among female participants. This alignment is quantified in Supplementary Table 5, which benchmarks our cohort against large-scale population data to support the external generalizability of our findings within the Asian context. However, a notable divergence was observed in our male cohort, which showed a higher prevalence of overweight status (68.5%) compared to the national averages of Taiwan (47.9%), Japan (58.9%), and Korea (61.7%).

### Model predictive performance

**Fig. 2:**
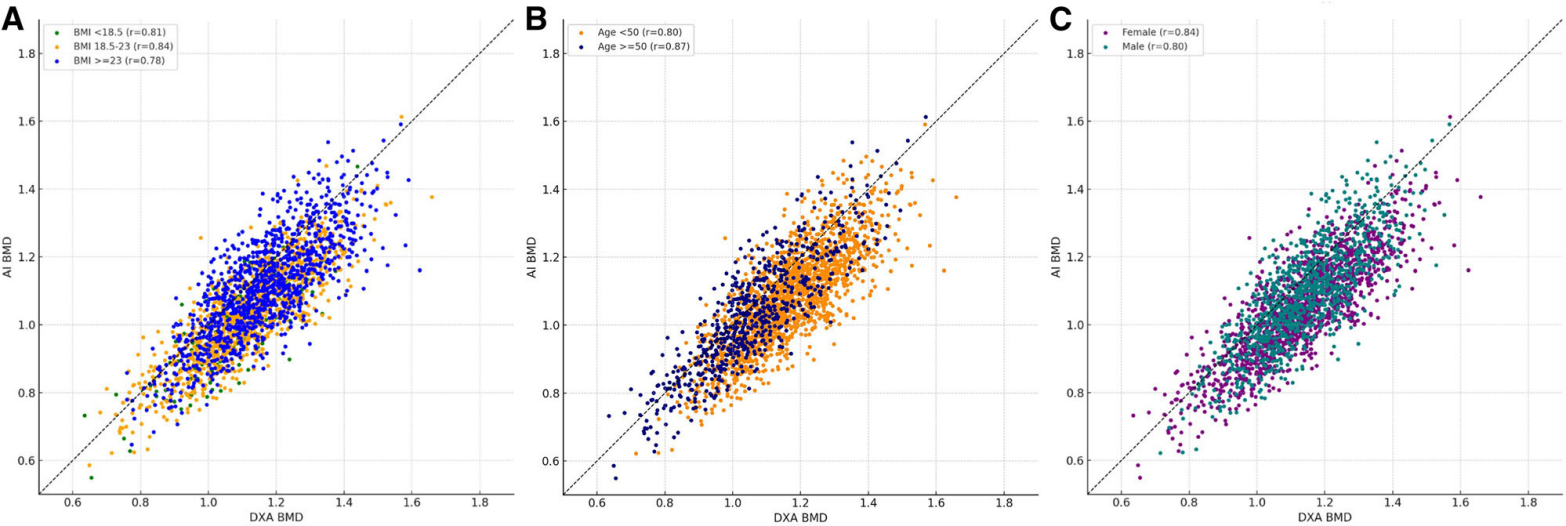
. Correlation between AI-predicted and DXA-measured bone mineral density across demographic subgroups. **A**–**C** Scatter plots show the relationship between AI-predicted bone mineral density (BMD; *y*-axis) and DXA-measured BMD (*x*-axis) in the validation cohort. Each point represents one participant. The dashed diagonal line indicates the line of equality (*y* = *x*). Panels are arranged from left to right as follows: **A** body mass index (BMI < 18.5, 18.5–23, ≥ 23 kg/m^2^), **B** age group (<50 and ≥50 years) **C** sex. Pearson correlation coefficients (*r*) are displayed within each panel.

The correlation between AI-predicted and DXA-measured bone mineral density (BMD) was evaluated across various physiological strata (Fig. 2A–C). Correlation coefficients remained generally consistent across categories defined by body mass index, ranging from 0.78 to 0.84, as well as across age groups (*r* = 0.80 for <50 years and *r* = 0.87 for ≥50 years) and sex (*r* = 0.84 for females and *r* = 0.80 for males). The identity plots illustrated that the predicted values were distributed close to the reference line across these groups, reflecting a stable linear relationship between the model outputs and the ground-truth measurements (Fig. 2A–C)

**Fig. 3:**
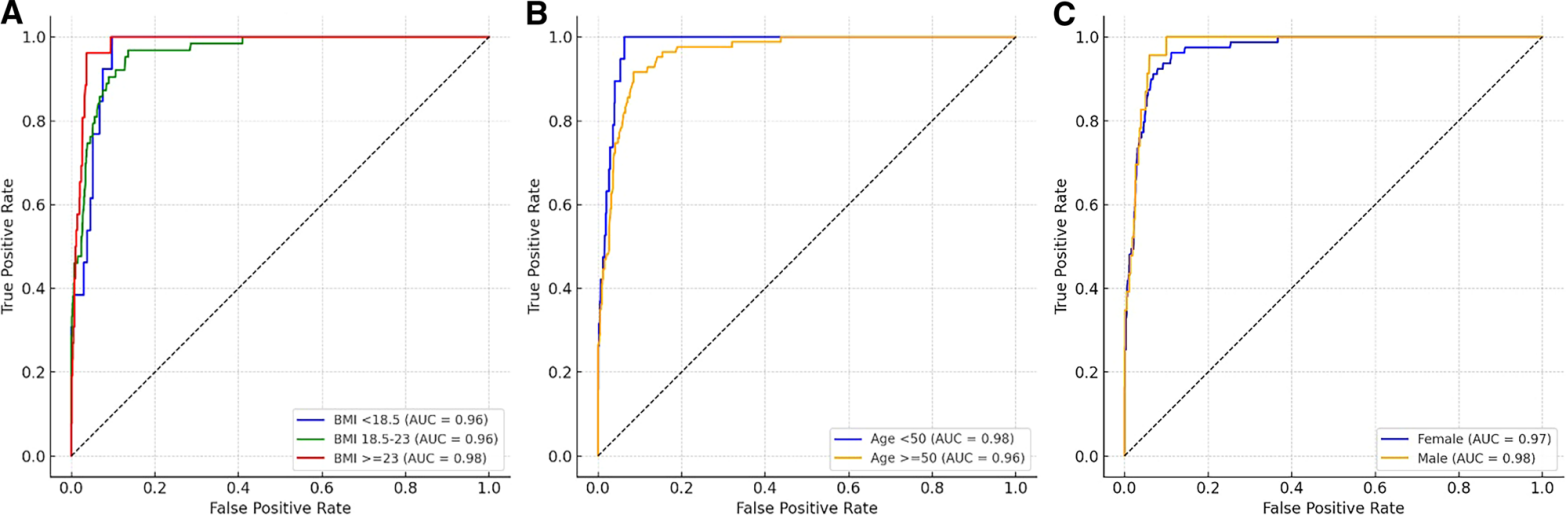
Receiver operating characteristic curves of AI-based detection of abnormal bone mineral density across demographic subgroups. **A**–**C** Receiver operating characteristic (ROC) curves show the performance of the AI model in identifying DXA-defined osteoporosis in participants aged ≥50 years (T-score ≤−2.5) and low bone mineral density for age in participants aged 50 years (Z-score ≤−2.0). The x-axis represents 1 − specificity and the y-axis represents sensitivity. The dashed diagonal line indicates chance-level discrimination (area under the curve [AUC] = 0.5). Panels are arranged from left to right as follows: **A** body mass index (BMI <18.5, 18.5–23, ≥23 kg/m^2^), **B** age group (<50 and ≥50 years), and **C** sex.

Receiver operating characteristic (ROC) analyses yielded high AUC values ranging from 0.96 to 0.98 across subgroups (Fig. 3A–C). For this population-level validation involving 118 DXA-confirmed positive cases (of which 94 were correctly identified by AI), these results provide a reliable estimation of model performance, with a sensitivity of 79.7% (95% CI: 71.3–86.5%) and an AUC of 0.95 (95% CI: 0.93–0.99) calculated via the Clopper–Pearson exact method. These intervals define the precision achievable with the current sample size and establish a baseline for interpreting subsequent exploratory subgroup analyses. However, as the analysis extends to the twelve intersectional sex–age–BMI strata, the resulting metrics should be interpreted as exploratory and hypothesis-generating because several cells contained only a small number of positive events. Supplementary Table 6 summarizes these subgroup-specific diagnostic metrics and explicitly illustrates the widening confidence intervals in strata with low disease prevalence.

### Distribution of DXA-confirmed true-positive cases

Among the 94 DXA-confirmed true-positive cases identified by the AI model, all presented with clinically significant abnormal bone status, defined as osteoporosis in participants aged ≥50 years or low bone mineral density for age in those aged <50 years. Supplementary Dataset 1 provides the anonymized clinical profiles for each of these 94 cases, detailing the specific T-scores or Z-scores that confirm the AI’s diagnostic accuracy. Females constituted the majority of the true-positive cohort (72/94, 76.60%), and more than half of all cases fell within the normal-weight BMI category of 18.5–23 kg/m² (56/94, 59.60%). Notably, women aged ≥50 years with a BMI of 18.5–23 kg/m² represented the single largest subgroup, accounting for 44.70% (42/94) of all confirmed cases.

Participants aged <50 years accounted for 19.10% (18/94) of the true-positive cases. Within this younger cohort, a notable sex-based divergence was observed, as the majority of cases occurred in men (13/18, 72.20%). These male cases were distributed across BMI categories of <18.5 kg/m² (*n* = 3), 18.5–23 kg/m² (*n* = 7), and ≥23 kg/m² (*n* = 4), whereas younger women contributed only five cases spanning all BMI strata. Among participants aged ≥50 years (*n* = 76), the gender distribution shifted significantly, with women constituting the majority (59/76, 77.60%) and predominantly clustering within the 18.5–23 kg/m² and ≥23 kg/m² BMI ranges.

True-positive cases were identified across all 12 sex, age, and BMI strata, with lumbar spine BMD values ranging from 0.635 to 0.880 g/cm². For participants aged ≥50 years, DXA T-scores ranged from −2.5 to −4.5, whereas for those aged <50 years, Z-scores ranged from −2.0 to −3.2. These individual-level characteristics, including precise densitometric indices, are detailed in Supplementary Dataset 1, reinforcing the model’s capacity to detect severe bone density loss across diverse physiological profiles.

### Subgroup diagnostic performance across sex, age, and BMI strata

Diagnostic performance was evaluated across 12 intersectional subgroups defined by sex, age, and BMI categories based on WHO Asia-Pacific criteria (Table 2). In the primary analysis of participants aged ≥50 years, where osteoporosis was defined by DXA T-scores ≤ –2.5, the model demonstrated high discriminative capacity with AUC values ranging from 0.93 to 0.97 in females and 0.96 to 1.00 in males; while the point estimates suggest exceptional discriminative capacity, the wider confidence intervals in smaller cohorts such as underweight men (BMI < 18.5 kg/m², *n* = 5) should be interpreted with caution. Sensitivity and negative predictive value (NPV) remained consistently robust across all elderly strata, ranging from 83.3% to 100.0% and 96.6% to 100.0%, respectively. Positive predictive value (PPV) in this age group showed greater variation, ranging from 37.5% to 80.0%, with the highest PPV observed in underweight women (BMI < 18.5 kg/m², 80.0%) and the lowest in men with BMI ≥ 23 kg/m² (37.5%).

**Table 2:** Diagnostic performance of the AI-assisted chest radiograph model across sex, age, and BMI subgroups

Subgroup/Metric	*N*	Sensitivity % (95% CI)	Specificity % (95% CI)	PPV % (95% CI)	NPV % (95% CI)	AUC (95% CI)
**≥50 years (Primary analysis)**						
Female, BMI < 18.5	24	100.0 (63.1–100.0)	87.5 (61.7–98.5)	80.0 (52.3–93.6)	100.0 (76.8–100.0)	0.95 (0.88–1.00)
Female, BMI 18.5–23	214	89.4 (76.9–96.5)	85.0 (78.7–90.1)	62.7 (53.6–71.0)	96.6 (92.5–98.5)	0.93 (0.89–0.97)
Female, BMI ≥ 23	171	94.4 (72.7–99.9)	90.9 (85.1–94.9)	54.8 (42.1–67.0)	99.3 (95.4–99.9)	0.97 (0.91–1.00)
Male, BMI < 18.5	5	100.0 (2.5–100.0)	75.0 (19.4–99.4)	50.0 (15.5–84.5)	100.0 (29.2–100.0)	1.00 (1.00–1.00)
Male, BMI 18.5–23	64	83.3 (35.9–99.6)	91.4 (81.0–97.1)	50.0 (28.7–71.3)	98.2 (89.8–99.7)	0.96 (0.85–1.00)
Male, BMI ≥ 23	169	100.0 (29.2–100.0)	97.0 (93.1–99.0)	37.5 (20.2–58.7)	100.0 (97.7–100.0)	0.99 (0.91–1.00)
**<50 years (Secondary analysis: low BMD for age)**						
Female, BMI < 18.5	99	100.0 (2.5–100.0)	85.7 (77.2–92.0)	6.7 (4.2–10.4)	100.0 (95.7–100.0)	0.95 (0.65–1.00)
Female, BMI 18.5–23	576	75.0 (19.4–99.4)	94.2 (92.0–96.0)	8.3 (4.5–14.9)	99.8 (99.0–99.9)	0.98 (0.88–1.00)
Female, BMI ≥ 23	286	100.0 (2.5–100.0)	97.2 (94.5–98.8)	11.1 (5.9–19.8)	100.0 (98.7–100.0)	0.99 (0.85–1.00)
Male, BMI < 18.5	19	100.0 (29.2–100.0)	81.3 (54.4–96.0)	50.0 (26.5–73.5)	100.0 (75.3–100.0)	0.94 (0.75–1.00)
Male, BMI 18.5–23	230	100.0 (54.1–100.0)	88.0 (83.0–91.9)	18.2 (13.5–24.0)	100.0 (98.1–100.0)	0.99 (0.93–1.00)
Male, BMI ≥ 23	478	100.0 (39.8–100.0)	96.4 (94.3–97.9)	19.1 (12.9–27.3)	100.0 (99.2–100.0)	0.98 (0.88–1.00)

Values are presented as percentages with 95% confidence intervals unless stated otherwise. Diagnostic metrics include sensitivity, specificity, positive predictive value (PPV), negative predictive value (NPV), and area under the receiver operating characteristic curve (AUC, DeLong method). Confidence intervals for proportions were calculated using exact (Clopper–Pearson) or Wilson methods based on sample size. Subgroups were defined by sex, age (<50 vs ≥50 years), and WHO Asia–Pacific BMI categories (<18.5, 18.5–23, ≥23 kg/m²). Osteoporosis was defined as T-score ≤ −2.5 for participants aged ≥50 years; low BMD for age was defined as Z-score ≤ −2.0 for participants aged <50 years. Subgroup analyses were exploratory, and estimates for strata with small denominators should be interpreted with caution.

In the exploratory analysis of participants aged <50 years (low BMD for age defined as Z-score ≤ −2.0), the tool maintained high discriminative capacity with AUC values between 0.94 and 0.99. NPVs were notably robust, exceeding 99.8% across all subgroups. However, PPVs exhibited a marked sex-based divergence in the younger cohort, with values remaining low among females (6.7–11.1%) compared to higher ranges observed in males (18.2–50.0%), particularly in those with BMI < 18.5 kg/m² (*n* = 19).

**Fig. 4:**
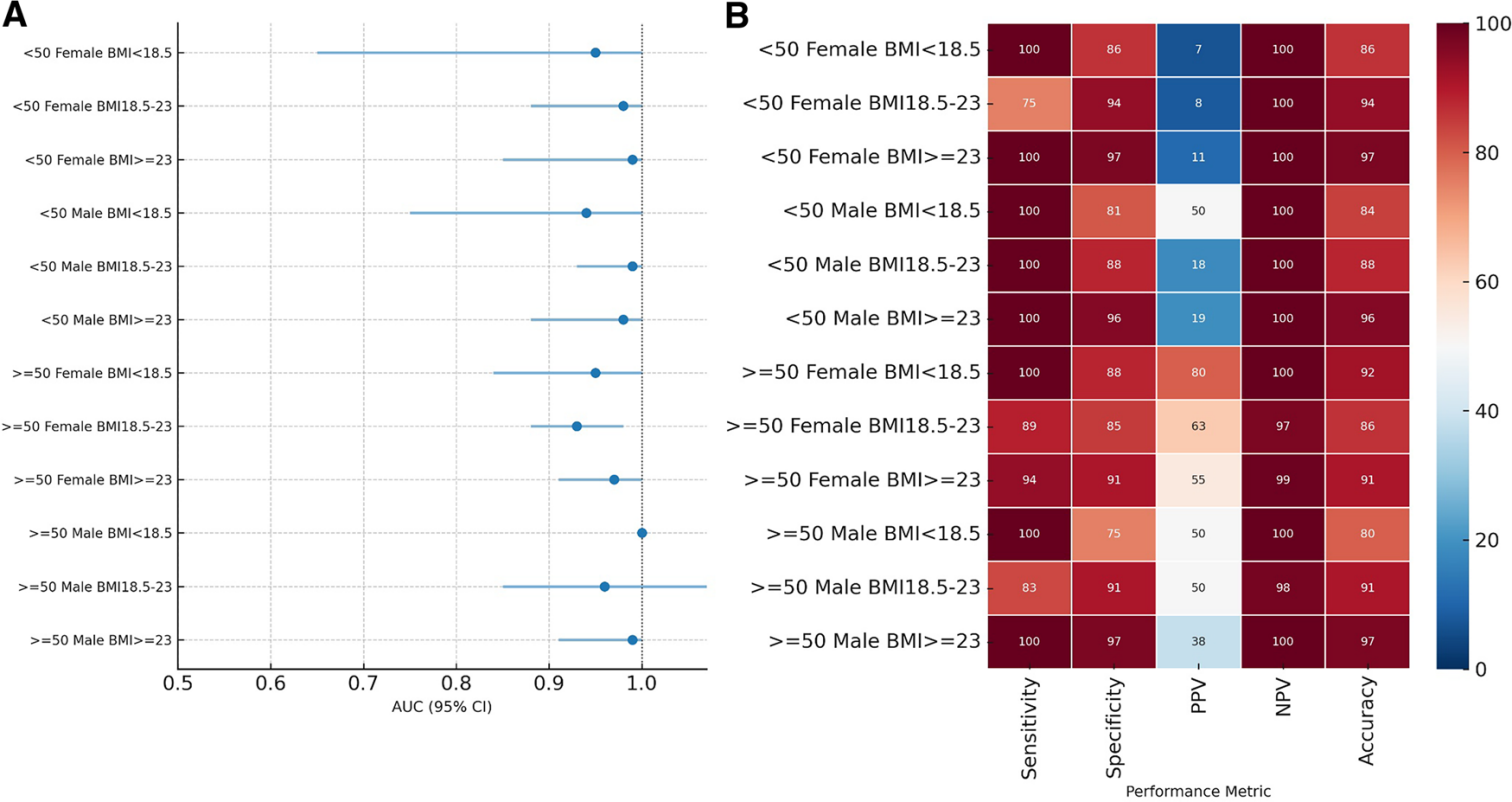
Subgroup diagnostic performance of the AI model across sex, age, and body mass index strata. **A** Forest plot showing area under the receiver operating characteristic curve (AUC) estimates with 95% confidence intervals for 12 intersectional subgroups defined by sex, age (<50 vs ≥50 years), and body mass index (BMI <18.5,18.5–23, ≥23 kg/m^2^). Error bars represent 95% confidence intervals estimated using the DeLong method. The dashed vertical line indicates chance-level discrimination (AUC = 0.5). **B** Heatmap summarizing subgroup-specific diagnostic performance, including sensitivity, specificity, positive predictive value (PPV), negative predictive value (NPV), and overall accuracy. Color intensity reflects the magnitude of each metric.

In the forest plot (Fig. 4A), AUC values demonstrated high discriminative capacity, ranging from 0.93 to 1.00 across all analyzed strata. Although 95% confidence intervals (CI) were wider in smaller cohorts, such as a 0.65–1.00 range observed in certain subgroups, the point estimates remained consistently elevated. Notably, the cohort of men aged ≥50 years with BMI < 18.5 kg/m² reached an AUC of 1.00 (95% CI 1.00–1.00), which was derived from a subgroup of five participants.

The heatmap (Fig. 4B) further illustrates that sensitivity and NPV remained robustly high, reaching up to 100% across most subgroups, whereas specificity and PPV exhibited greater variation. In participants aged ≥50 years, PPV ranged from 37.5% to 80.0%, with the highest value recorded in underweight females with a BMI < 18.5 kg/m². Conversely, in the exploratory analysis of participants aged <50 years, while AUCs remained high between 0.94 and 0.99 and NPVs exceeded 99.8%, PPVs showed a marked sex-based divergence. Specifically, PPV values were lower among female subgroups (6.7–11.1%) compared to male subgroups (18.2–50.0%). Overall, the tool exhibited consistent rule-out capability across all strata, with performance fluctuations in PPV and specificity aligning with the varying clinical profiles and disease prevalence across age and sex categories.

Subgroup-specific diagnostic performance and prevalence-standardized predictive values were further evaluated to assess the model’s clinical utility under diverse epidemiological contexts. These standardized metrics, detailed in Supplementary Table 6, provide an estimate of the model’s Positive Predictive Value (PPV) and Negative Predictive Value (NPV) across varying prevalence levels, illustrating its potential impact when deployed in populations with higher or lower disease burdens than our current cohort. Observed disease prevalence varied substantially across the intersectional strata, ranging from minimal levels between 0.35% and 2.61% in younger participants (aged <50 years) within the normal-weight (18.5–23 kg/m²) and overweight/obese (≥23 kg/m²) BMI strata, to 33.33% in underweight women (aged ≥50 years, BMI < 18.5 kg/m²). Correspondingly, positive likelihood ratios (LR+) spanned from 4.00 to over 35.00, while negative likelihood ratios (LR−) remained near zero across most strata, indicating a strong potential for ruling out the disease.

To account for population-level variations, standardization was performed using Bayes’ theorem at plausible prevalence levels derived from East Asian community data, specifically 7% for men and both 10% and 25% for women. These standardized analyses demonstrated that positive predictive values (PPV_std) improved substantially under higher prevalence scenarios, whereas negative predictive values (NPV_std) remained robustly high across all assumed levels. Furthermore, indices of screening efficiency and workload showed that the number of DXA examinations required per detected case ranged from 1.08 to 4.32. Lower efficiency, characterized by a higher number of scans required per detection, was consistently observed in strata with lower disease prevalence. These descriptive analyses demonstrate the direct relationship between population prevalence and the anticipated yield or workload of AI-assisted screening across different clinical scenarios.

### Calibration and decision curve analysis

**Fig. 5:**
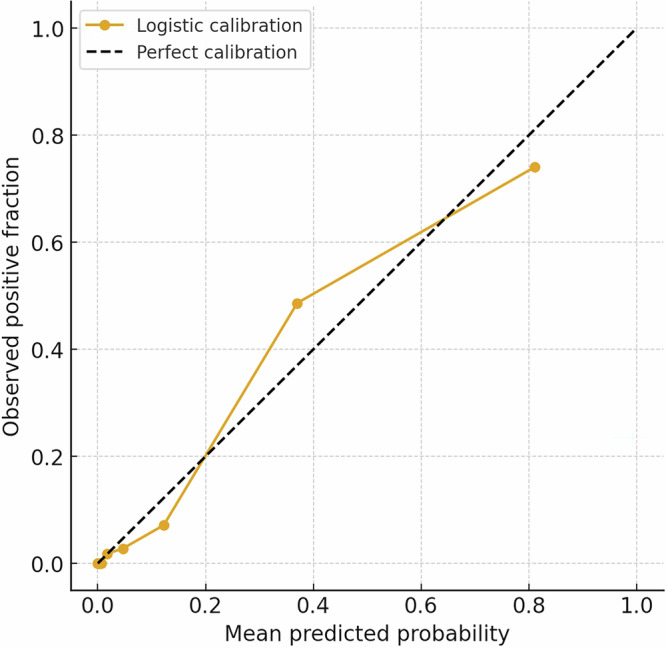
Calibration of AI-predicted osteoporosis risk in participants aged ≥50 years. Calibration plot comparing predicted probabilities with observed prevalence of DXA-defined osteoporosis (T-score ≤−2.5). Predicted risks were grouped into deciles. Points represent mean predicted probabilities and observed event rates within each decile. The dashed diagonal line indicates perfect calibration.

Calibration analysis demonstrated that the predicted risk probabilities were highly consistent with the observed prevalence of osteoporosis among participants aged 50 years or older (Fig. 5). The calibration curve closely aligned with the diagonal reference line across most of the risk spectrum, which indicates high reliability in risk estimation, particularly within the low-to-moderate range. While slight overestimation occurred at the highest risk decile, where the sample size was more limited, quantitative metrics confirmed robust performance with a Brier score of 0.051, a calibration intercept of 0.068, and a calibration slope of 1.089. These results were achieved using a fixed operating threshold based on the standard WHO criterion of a DXA T-score of −2.5 or lower without post-hoc optimization, a factor that supports the generalizability of the model’s predictions (Fig. 5).

**Fig. 6:**
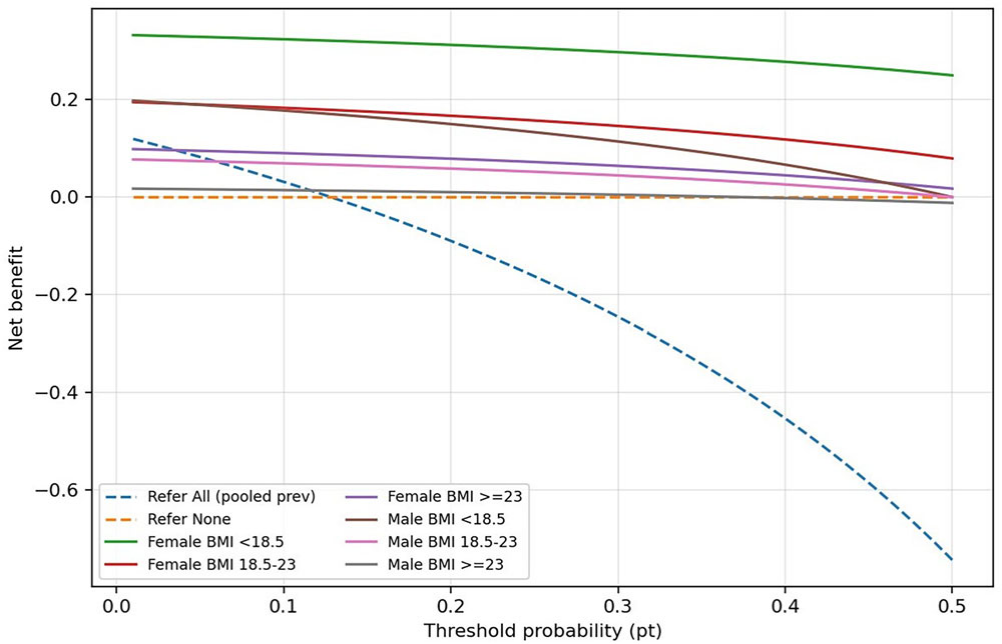
Decision curve analysis of AI-assisted referral for DXA in participants aged ≥50 years. Decision curve analysis compares the net clinical benefit of the AI-assisted referral strategy with default strategies of referring all participants (“refer all”) or referring none (“refer none”) across a range of threshold probabilities. Net benefit was calculated as described in the “Methods.” The “refer all” strategy was calculated using the overall (pooled) prevalence of DXA-defined osteoporosis in this cohort (“pooled prev” in the figure). Curves are shown for subgroups stratified by sex and body mass index (BMI<18.5, 18.5–23, ≥23 kg/m^2^).

Decision curve analysis (DCA) substantiated the clinical utility of the AI-based referral strategy across a broad range of threshold probabilities (0–50%) (Fig. 6). The AI model consistently provided a higher net benefit compared to the default strategies of “refer all” or “refer none,” with the most substantial clinical gains observed between thresholds of 5% and 30%.

Stratified DCA for participants aged ≥50 years revealed that the magnitude of net benefit was highly dependent on sex and BMI profiles. The clinical utility was most pronounced in women, especially those in the underweight (BMI < 18.5 kg/m²) and normal-weight (BMI 18.5–23 kg/m²) categories, whose curves remained well above the “refer all” baseline. In contrast, for men with BMI ≥ 23 kg/m², the net benefit remained relatively marginal, approaching the “refer none” strategy. These intersectional patterns suggest that while the AI tool provides a universal benefit, its impact on reducing unnecessary DXA scans is most significant in high-risk physiological strata (Fig. 6).

## Discussion

Osteoporosis remains a major public health concern, affecting more than half of adults aged 50 years and older and contributing to substantial morbidity and mortality worldwide^29^. Low-trauma osteoporotic fractures are associated with increased long-term mortality and a high risk of subsequent fractures. This underscores the importance of early identification and prevention^30^. Yet screening continues to be underutilized, particularly among men, younger adults (<50 years), and individuals with normal BMI (18.5–23 kg/m², per WHO Asia-Pacific criteria). Current guideline-based approaches predominantly target women aged ≥50 years with BMI < 18.5 kg/m² or other high-risk features and may inadvertently overlook these populations.

In this study, an AI-CXR opportunistic screening strategy using routine chest radiographs demonstrated favorable diagnostic performance in a preventive health cohort. The model achieved high discrimination (AUCs 0.93–1.00) and high negative predictive value across all 12 intersectional subgroups defined by sex, age (<50 vs. ≥50 years), and WHO Asia–Pacific BMI categories (Fig. 4; Table 2). Performance was maintained even in smaller strata such as men aged ≥50 years with BMI < 18.5 kg/m² and women aged <50 years with BMI ≥ 23 kg/m². These groups are rarely represented in osteoporosis screening research (Supplementary Table 6). Subgroup analyses were exploratory. Estimates from strata with small denominators, particularly among adults aged <50 years or specific combinations of age, sex, and BMI, should be interpreted descriptively rather than inferentially. Although individuals aged <50 years with low BMD for age (Z-score ≤ −2.0) were included in secondary analyses, the clinical implications differ from those in older adults. Such findings may warrant further evaluation, but do not indicate osteoporosis or necessitate the same management approach.

These findings are consistent with the emerging literature on AI-CXR for osteoporosis case-finding and build on prior prospective work. In the OPSCAN randomized trial, AI-CXR triage increased downstream DXA utilization and new osteoporosis diagnoses among high-risk individuals in a referral population^20^. By contrast, the present study evaluated AI-CXR in a lower-risk community health-check cohort with primary care settings. This offers complementary evidence in preventive contexts differing from specialty referrals, where baseline risk, referral pathways, and DXA access differ substantially from specialty clinics.

To our knowledge, this study represents the first real-world validation of AI-CXR explicitly stratified across WHO Asia–Pacific BMI categories in an Asian preventive health cohort. A substantial proportion of DXA-confirmed osteoporosis cases occurred among individuals with normal BMI (18.5–23 kg/m²), particularly older women(age ≥50 years). This indicates that body weight alone does not guarantee skeletal protection. This highlights the limitations of using BMI as a sole proxy for bone health in Asian populations. It exposes a diagnostic blind spot in current screening paradigms. Over-reliance on BMI heuristics may perpetuate underdiagnosis not only among BMI < 18.5 kg/m² and BMI ≥ 23 kg/m² individuals but also within ostensibly “normal”(18.5–23 kg/m²) populations^31^. These observations align with recent meta-analyses showing heterogeneous bone outcomes across the BMI spectrum. Obesity does not uniformly protect against osteoporosis^32^.

From a population perspective, the BMI and sex–age distribution of this cohort closely mirrored those reported in large East Asian surveys, including Taiwan’s NAHSIT 2017–2020, Japan’s 2022 National Health and Nutrition Survey, and Korea’s KNHANES. These surveys collectively document high overweight prevalence in men and persistent underweight among younger women^33–35^. These regional consistencies support the broader applicability of the findings. They suggest that AI-CXR may generalize to other East Asian populations with comparable anthropometric profiles^33–35^.

This study has several strengths and limitations that should be considered when interpreting the findings. Strengths include the use of systematically collected DXA as a reference standard in a real-world preventive health cohort, comprehensive analyses across intersectional subgroups of age, sex, and BMI, and the evaluation of discrimination, calibration, and decision-analytic net benefit using prevalence-standardized predictive values. Several limitations must also be acknowledged. First, this was a retrospective study with a relatively low prevalence of abnormal BMD (3.9%), reflecting the nature of an asymptomatic health-check population. Given the low underlying prevalence of DXA-positive cases in this asymptomatic cohort (3.9%), a modest PPV is mathematically expected, even for a model with high sensitivity and reasonable specificity. This aligns with the intended role of the AI-CXR tool as a triage mechanism to prioritize individuals for DXA confirmation, rather than as a definitive diagnostic test. While the absolute number of 94 DXA-confirmed positive cases may limit the statistical power for complex intersectional subgroup analyses, we reported precise 95% confidence intervals of 71.3–86.5% for sensitivity and 0.93–0.99 for AUC to transparently characterize statistical uncertainty. The robustness of these performance estimates, despite the modest event count, underscores the model’s reliability in low-prevalence screening environments. Nevertheless, we addressed this by focusing on descriptive rather than inferential interpretations for the smaller strata. Second, the study was hospital-based and conducted within a single health system, which may limit generalizability; external validation across other populations and healthcare systems, including community-based and multi-ethnic cohorts, is warranted. Third, the reference standard DXA has inherent limitations. For instance, in older adults, lumbar spine BMD measurements can be artifactually elevated by osteophytes or other severe degenerative changes, potentially leading to an underestimation of osteoporosis risk. Although we applied standardized exclusion criteria for factors such as inadequate image quality, spinal hardware, or infections, these elements may still influence generalizability. Specifically, our stringent DXA inclusion criteria—requiring all four lumbar vertebrae (L1–L4) to be evaluable and excluding scans with a T-score difference >1.0 between adjacent vertebrae—may have inadvertently excluded patients with focal structural pathologies, such as prevalent vertebral fractures, severe scoliosis, or pronounced degenerative changes. These conditions often cause the very intervertebral heterogeneity that triggered exclusion in our protocol. Consequently, our findings primarily represent the model’s performance in structurally homogeneous spines and may not generalize to patients with established vertebral deformity or spinal hardware. Intriguingly, this discrepancy may also signify a latent strength of deep learning because AI analysis of radiographs might offer a more nuanced interpretation of bone quality by accounting for structural interferences that often artifactually inflate DXA values. This hypothesis warrants further investigation using 3D-CT or fracture outcomes.

Fourth, our retrospective selection approach could introduce sampling bias because inclusion was limited to participants with available chest radiographs within six months of DXA. Furthermore, femoral neck or total hip BMD values were not available in this dataset, which precluded hip-based sensitivity analyses. Finally, the lack of detailed clinical risk factors such as menopausal status, fracture history, and glucocorticoid use limited the clinical depth of our analysis. Because these variables were not collected, we could not fully categorize the study population according to standard clinical risk profiles or assess how the AI model interacts with known predictors of bone loss. Consequently, while exploratory analyses using conservative proxy criteria such as age, sex, and BMI indicated that 28.7% or fewer AI-flagged individuals met proxy ISCD eligibility, these findings remain constrained by the incomplete clinical phenotyping. These limitations emphasize the need for future prospective studies that incorporate comprehensive clinical data and longitudinal outcomes to better define the role of AI in integrated screening pathways.

From a health systems perspective, opportunistic AI-CXR represents a pragmatic, infrastructure-light approach. Since chest radiographs are already widely performed for preventive and clinical purposes, adding AI incurs negligible marginal cost or patient burden. Unlike DXA, which is constrained by equipment availability and referral practices, AI-CXR serves as an automated triage tool to prioritize individuals who might otherwise be overlooked by traditional age-based or risk-score-based screening. This approach could potentially reduce missed diagnoses and optimize resource use, particularly in regions with low DXA penetration. While these results are encouraging for population-level screening, they should not be directly extrapolated to high-risk groups, such as patients in fracture clinics or those on long-term glucocorticoid therapy. For such populations, our findings serve as a hypothesis-generating foundation that necessitates further real-world, prospective evaluation.

Beyond regulatory approval, real-world integration requires careful validation and transparent implementation. The evaluated Software as a Medical Device (VeriOsteo™ OP) has received regulatory clearance in several Asian jurisdictions, supporting feasibility for regional deployment. Nonetheless, clinical adoption remains contingent on country-specific regulatory frameworks, workflow integration, and demonstration of long-term clinical impact. Lessons from other healthcare AI applications, including over-reliance, confirmation bias, and automation complacency, underscore the need for transparency, human-in-the-loop verification, and continued regulatory oversight in any deployment^36^.

This work also contributes to the growing literature on algorithmic fairness. By retraining the model on representative local data and validating across intersectional strata of sex, age, and BMI, the study illustrates an approach to mitigating bias and promoting equitable access, in contrast to cautionary examples where unchecked AI deployment has amplified health inequities. Thus, AI integration serves as a technical lever to operationalize diagnostic equity in clinical practice. In preventive health settings where structured risk factor documentation is often incomplete, AI-CXR can identify at-risk individuals without relying on extensive clinical history, further underscoring its potential utility for large-scale opportunistic screening. It also highlights the importance of transparent communication about model limitations and uncertainty.

At a regional level, these findings align with consensus efforts such as the Asia–Pacific Consortium on Osteoporosis (APCO) Framework. This advocates harmonized minimum standards of care, earlier diagnosis, and equitable access to fracture prevention across the Asia–Pacific region^37^. They also resonate with the International Osteoporosis Foundation’s call to close the “osteoporosis care gap” ^38^ through earlier identification, context-appropriate screening strategies, and integration of innovative technologies into routine care, as well as with the World Health Organization’s Global Strategy on Digital Health 2020–2025, which emphasizes the role of digital tools, including AI, in strengthening health systems^39^.

Future research should focus on prospective, multi-ethnic, and community-based validation to confirm transportability and clinical utility across diverse populations. Randomized or pragmatic trials are needed to evaluate real-world clinical outcomes, including fracture incidence, treatment initiation, adherence, and recurrence, as well as the impact on workflow and clinician trust. Formal cost-effectiveness analyses will be essential to quantify the value of AI-CXR triage relative to existing case-finding strategies in different health system contexts. In parallel, integration of comprehensive clinical risk factors and longitudinal data into AI models, coupled with robust governance frameworks, will be critical. This ensures safe, equitable, and sustainable deployment within national preventive health programs, consistent with international digital health strategies^39^.

## Methods

### Study design, setting, and ethical considerations

We conducted a retrospective, cross-sectional study to evaluate the real-world performance of VeriOsteo™ OP, a deep learning model developed by Taichung Veterans General Hospital (TCVGH, https://www.vghtc.gov.tw) and Acer Medical Inc., for opportunistic osteoporosis screening using posteroanterior (PA) chest radiographs (CXRs). The AI model was originally trained and internally validated using paired CXR-DXA data from TCVGH, a tertiary referral center in central Taiwan. For external validation, we used data from 2384 asymptomatic adults who underwent self-paid health check-ups at the health management center of St. Paul’s Hospital, a regional teaching hospital in northern Taiwan, between 1 January 2012 and 6 November 2023.

Eligible participants were aged ≥20 years and had both a digital PA chest radiograph (resolution ≥1024 × 1024 pixels) with adequate thoracolumbar visualization and a lumbar DXA scan within ±6 months. Exclusion criteria included spinal implants, severe deformities, malignancy, or infection near the T12-L1 region. All CXRs were acquired using a source-to-image distance of 180 cm, tube voltage 70–124 kVp, and exposure 2–15 mAs, ensuring adequate thoracic cavity inclusion and sufficient exposure. Images not meeting these quality criteria were excluded. CXRs that did not meet these quality-control criteria (e.g., incomplete visualization of the thoracolumbar junction, motion or exposure artifacts, or metallic implants) were categorized as ‘unevaluable’ and excluded from analysis.

All clinical and imaging data were de-identified and extracted from electronic health records. The study protocol was approved by the Institutional Review Board of Taichung Veterans General Hospital (IRB No. SE23538A), with a waiver of informed consent due to the retrospective use of anonymized secondary data.

BMI was categorized according to WHO Asian thresholds: underweight (<18.5 kg/m²), normal (18.5–23 kg/m²), and overweight (≥23 kg/m²). Our cohort’s BMI and sex distribution closely matched population data from Taiwan (NAHSIT), Japan (NHNS), and South Korea (KNHANES), suggesting regional generalizability (Supplementary Table 5). Image quality was verified by the principal investigator, and AI inference was conducted using VeriOsteo™ OP, which outputs continuous BMD estimates and binary classifications for suspected abnormal BMD (saBMD).

### AI system and imaging procedure

We evaluated the VeriOsteo™ OP software (Acer Medical Inc., New Taipei City, Taiwan), an AI-assisted tool for opportunistic osteoporosis screening from chest radiographs. The system follows a locked-model design and comprises two modules: (1) a vertebral landmark detection module that identifies the lowest thoracic and first lumbar vertebrae, and (2) a deep learning model for BMD estimation based on the Vision Transformer (ViT-Large) architecture.

During training, input chest X-ray images were resized to 448 × 448 pixels and partitioned into non-overlapping 16 × 16 patches, which were processed by a Transformer encoder (24 layers) followed by a multilayer perceptron (MLP) head to predict BMD. The model was trained on paired chest radiograph-DXA data, with DXA-derived L1–L4 BMD serving as the reference standard. Model outputs included both a continuous BMD estimate and a binary classification of suspected abnormal BMD, mapped to T- or Z-score thresholds based on age.

The system operates fully autonomously without the need for clinical metadata or manual image curation. All model parameters are fixed prior to deployment, and the software does not permit user-side retraining or parameter tuning. VeriOsteo™ OP has obtained regulatory approval as a medical device in Taiwan, Singapore, Thailand, Malaysia, and Indonesia, supporting the feasibility of deployment in real-world healthcare settings subject to local requirements. Detailed model architecture and preprocessing steps are summarized in the model card (Supplementary Table 8).

### Bone mineral density reference standard

The AI-CXR tool was utilized to flag suspected abnormal bone mineral density (saBMD) for referral. In this study, abnormal BMD (the reference standard) was defined as osteoporosis for participants aged ≥50 years, using the WHO 1994 threshold of T-score ≤ −2.5, and as low bone mineral density for age in participants aged <50 years, defined by the ISCD Official Positions (Z-score ≤ −2.0, below the expected range for age). In line with ISCD guidance, osteoporosis was not diagnosed solely on BMD in men under 50 years or in children.

Dual-energy X-ray absorptiometry (DXA) of the lumbar spine (L1–L4) served as the diagnostic reference standard. All scans were performed on a GE Lunar Prodigy Advance densitometer (GE HealthCare, Madison, WI, USA) using enCORE software version 18, with daily phantom calibration according to manufacturer protocols. Scans were considered evaluable only if all four lumbar vertebrae were included and the absolute difference in T-scores between adjacent vertebrae did not exceed 1. DXA records that did not meet these criteria or had missing data were excluded from analysis.

T-scores were calculated from the measured bone mineral density (BMD, g/cm²) using the mean and standard deviation of young Caucasian women aged 20–29 years from the Third National Health and Nutrition Examination Survey (NHANES III) as the reference population, in accordance with World Health Organization (WHO) and International Society for Clinical Densitometry (ISCD) positions. Z-scores were derived from age-, sex-, and ethnicity-matched reference values.

The precision error of lumbar spine DXA at St. Paul’s Hospital, assessed by phantom and repeat patient scans, corresponded to a coefficient of variation (CV) of <1.5%, consistent with international quality-control standards. Further details of the DXA reference database and system calibration are provided in Supplementary Table 9 and Supplementary Fig. 1.

### Model evaluation and performance metrics

We assessed AI model performance on the independent external validation cohort, using the internal test set from development as a benchmark. Following prior validation studies, we adopted the area under the receiver operating characteristic curve (AUC) as the primary metric. Secondary performance measures included accuracy, sensitivity, specificity, positive predictive value (PPV), and negative predictive value (NPV), calculated against DXA-confirmed ground truth. Agreement between AI-predicted and DXA-measured BMD was evaluated using Pearson’s correlation coefficient, and scatter plots were generated for visualization.

Group differences in performance metrics (e.g., sensitivity, specificity) were tested using two-proportion z-tests. Comparisons of demographic characteristics between the development and validation cohorts used Student’s *t*-tests for continuous variables and χ^2^ tests for categorical variables.

### Subgroup analyses for model generalizability

To assess model generalizability and fairness, we performed predefined subgroup analyses on the validation dataset. Screening performance was stratified by sex (female vs male), age (<50 vs ≥50 years), and WHO-defined BMI categories for Asian populations (<18.5, 18.5–23, ≥23 kg/m²). For each subgroup, AUC and other diagnostic metrics were calculated, with 95% confidence intervals reported. Statistical tests were applied to evaluate whether sensitivity or specificity differed across subgroups. These analyses aimed to ensure that the model performed equitably across diverse demographic and anthropometric characteristics. No correction for multiple testing was applied, as these analyses were exploratory and hypothesis-generating.

### Calibration analysis

The AI model generated predicted T-scores, reflecting deviations from the young-adult reference mean. Calibration was evaluated using logistic calibration based on the difference from the diagnostic threshold (Δ = *T* + 2.5).

Predicted probabilities were stratified into 10 equally sized bins (quantile binning). Within each bin, the mean predicted risk was compared with the observed osteoporosis prevalence, and the results were plotted against the 45° diagonal. Calibration performance was quantified using the Brier score, as well as the intercept and slope estimated from logistic regression. An intercept of 0 and a slope of 1 indicate perfect calibration. The diagnostic threshold (T-score ≤ −2.5) was prespecified according to WHO criteria and not tuned on this dataset.

### Model calibration and decision curve analysis

Calibration was assessed using quantile-binned reliability curves, the Brier score, and a logistic calibration model that mapped AI-predicted T-scores to risk probabilities; calibration intercept and slope are reported. The Brier score was computed as the mean squared difference between predicted probabilities and observed outcomes:

$$\mathrm{Brier}=\frac{1}{{\rm{N}}}\mathop{\sum }\limits_{{\rm{i}}=1}^{N}{\left({{\rm{p}}}_{{\rm{i}}}-{{\rm{o}}}_{{\rm{i}}}\right)}^{2}$$

Decision curve analysis (DCA) was used to quantify net clinical benefit across threshold probabilities of 5–30%, prespecified as clinically relevant for opportunistic screening. Net benefit at each threshold was calculated as (TP/N) − (FP/N) × [pt/(1 − pt)], where pt is the decision threshold probability at which a clinician would opt to refer a patient for DXA^40^. The classification threshold for defining osteoporosis was fixed a priori according to the WHO definition (T-score ≤ −2.5) and was not tuned on this dataset.

### Standardization of predictive values

Because positive and negative predictive values (PPV and NPV) depend on disease prevalence, standardized PPV and NPV were derived using Bayes’ theorem (equivalently, from likelihood ratios and a fixed prior) for adults aged ≥50 years. Population-plausible prevalences of DXA-defined osteoporosis (T-score ≤ −2.5) were assumed—25% for women and 7% for men—based on community-representative data from East Asia^31^. Sensitivity analyses using alternative prevalence assumptions (women 20–35%; men 5–10%) are presented in Supplementary Table 6. For participants aged <50 years, osteoporosis is not formally defined by T-score; therefore, PPV/NPV estimates are reported descriptively and were not standardized to population prevalence.

### Statistical analysis

Baseline characteristics of the study population were summarized using descriptive statistics. Continuous variables were reported as mean ± standard deviation or median with interquartile range (IQR), and categorical variables were presented as counts and percentages. Group comparisons were performed using Student’s *t*-test, Wilcoxon rank-sum test, or χ^2^ test as appropriate.

The diagnostic performance of the AI model was evaluated using sensitivity, specificity, and positive predictive value (PPV). Specifically, sensitivity was calculated as the proportion of AI-detected true positives (*n* = 94) among all DXA-confirmed abnormal BMD cases (*n* = 118). To ensure statistical rigor, 95% confidence intervals (CIs) for all proportional metrics were calculated using the Clopper–Pearson exact binomial method. For the Area Under the Receiver Operating Characteristic curve (AUC), 95% CIs were estimated via the DeLong method.

Given the retrospective design, no formal sample size calculation was performed. The identification of 118 DXA-positive individuals allowed for robust estimation of population-level performance. However, when stratified into subgroups by sex, age, and BMI categories, the limited number of positive cases per stratum resulted in wider confidence intervals. These subgroup-level estimates should therefore be interpreted as exploratory.

All statistical tests were two-sided, with a significance level of *p*-values < 0.05.

Analyses were performed using Python (version 3.11) and MedCalc Software Ltd. (version 23.3.7, Ostend, Belgium), with standard libraries for ROC analysis, confidence interval estimation, and calibration.

## Supplementary information

Supplementary Information

## Data Availability

The datasets generated and/or analyzed during the current study are de-identified, but due to patient data confidentiality and Institutional Review Board requirements, they are not publicly available. They can be obtained from the corresponding author upon reasonable request.

## Code availability

The custom code used for this study is proprietary to Acer Medical Inc. and cannot be made publicly available due to company confidentiality and intellectual property restrictions. The code was implemented using Python (version 3.12) and C# (version 7.3). Specific variables and parameters used for model training and statistical analysis are described in the “Methods” section and Supplementary Information, providing sufficient information for independent reproduction of the results.
