# Supplementary material for: Advancing diagnostic equity through artificial intelligence chest radiograph screening for osteoporosis in Asian populations
Source: NPJ Digit Med. 2026 Mar 19;9:359. doi: 10.1038/s41746-026-02484-x (PMC13153408; doi:10.1038/s41746-026-02484-x)

Supplementary Table 1. Summary of major osteoporosis screening guidelines and eligibility criteria

Guideline (Source)	Year (Publication)	Primary Target Population for Screening	Key Screening Criteria or Definition	Major Limitations / Coverage Gaps
**National Osteoporosis Foundation (NOF)** — Clinician's Guide to Prevention and Treatment of Osteoporosis (*Osteoporos Int.* 2014; 25: 2359–2381) [Cosman et al., 2014]	2014	Postmenopausal women and men aged ≥ 50 years with clinical risk factors or fragility fracture history	Defines osteoporosis as T-score ≤ –2.5 at the femoral neck, total hip, or lumbar spine (by WHO criteria). Recommends BMD testing for women ≥ 65 y, men ≥ 70 y, and younger individuals with risk factors.	Primarily clinical risk-based; does not support population-level screening or address sex/age equity issues.
**International Society for Clinical Densitometry (ISCD)** — Official Positions on Adult and Pediatric DXA Testing (https://iscd.org, updated 2023) [ISCD, 2023]	2023	Adults ≥ 50 years (old) for T-score assessment; younger (<50 y) evaluated using Z-score relative to age/sex/ethnicity	Osteoporosis defined as T ≤ –2.5 for ≥ 50 y; for < 50 y, "low BMD for age" if Z ≤ –2.0. Recommends DXA for postmenopausal women and men with clinical risk factors or secondary causes.	Screening based on clinical referral or indication; no guidance for opportunistic imaging or AI-assisted approaches.
**US Preventive Services Task Force (USPSTF)** — Screening for Osteoporosis to Prevent Fractures (*JAMA* 2025; doi: 10.1001/jama.2024.27154) [USPSTF, 2025]	2025	Women ≥ 65 years; postmenopausal women < 65 y with risk factors; men — insufficient evidence (I statement)	Recommends central DXA BMD testing with or without FRAX-based risk assessment for eligible women. Finds insufficient evidence for screening men.	Continues to exclude men and younger adults from routine screening; highlights ongoing equity gap and need for new risk-assessment strategies.

Supplementary Table 2. Baseline Characteristics by Age

Variable	**Overall (n = 2384)**	Age < 50 N=1722	Age ≥ 50 N=662	
Sex				
Male, n (%)	1008 (42.3%)	761 (44.2%)	247 (37.3%)	
Female, n (%)	1376 (57.7%)	961 (55.8%)	415 (62.7%)	
Age, mean ± SD, years	43.6 ± 9.9(20-99)	40.4 ± 6.3	56.2 ± 4.8	
BMI, mean ± SD, kg/m²	23.4 ± 3.9	23.0 ± 3.8	24.3 ± 4.1	p<0.0001
BMI <18.5, n (%)	223(9%)	99 (5.8%)	48 (7.3%)	
BMI 18.5–23, n (%)	1457(61%)	810 (47.0%)	247 (37.3%)	
BMI ≥23, n (%)	704(30%)	813 (47.2%)	367 (55.4%)	
Bone Mass Density				
BMD, mean ± SD, g/cm²	1.13 ± 0.17	1.16 ± 0.13	1.08 ± 0.18	p<0.0001
T-score, mean ± SD	-0.33 ± 1.24	-0.15 ± 1.14	-0.80 ± 1.27	p<0.0001
Z-score, mean ± SD	0.44 ± 1.13	0.56 ± 1.10	0.09 ± 1.13	
Suspected abnormal BMD (saBMD), n (%)	255 (11%)	129 (7.5%)	126 (19.0%)	p<0.0001

Variable	**Overall (n = 2384)**	Age < 50<br>N=1722	Age ≥ 50<br>N=662
Sex			
Non-saBMD, n (%)	2129 (89%)	1593 (92.5%)	536 (81.0%)
Collected images			
Evaluable CXRs, n (%)	2335 (98%)	1698 (98.6%)	637 (96.2%)
Not Evaluable CXRs, n (%)	49 (2%)	24 (1.4%)	25 (3.8%)

Data are presented as n (%) for categorical variables and mean ± standard deviation (SD) for continuous variables. saBMD = suspected abnormal bone mineral density (defined as T-score ≤ –2.5 for participants aged ≥ 50 years, or Z-score ≤ –2.0 for participants aged < 50 years); BMD = bone mineral density; CXR = chest X-ray. P-values were calculated using Student's t-test for continuous variables and Chi-square test for categorical variables. Evaluable CXR refers to images meeting predefined quality-control criteria (adequate thoracolumbar visualization, absence of artifacts or implants), whereas Not Evaluable CXR indicates images excluded for insufficient quality. These descriptive findings illustrate significant sex differences in BMI distribution, saBMD prevalence, and the proportion of evaluable chest radiographs, highlighting the importance of subgroup analyses in evaluating AI model performance and health equity.

Supplementary Table 3. Baseline Characteristics by BMI

Variable	**Overall (n = 2384)**	BMI <18.5<br>N=147	BMI 18.5–23<br>N=1094	BMI ≥23<br>N=1143

Sex				
Male, n (%)	1008 (42.3%)	33 (22.4%)	146 (13.5%)	69 (6.2%)
Female, n (%)	1376 (57.7%)	114 (77.6%)	938 (86.5%)	1035 (93.8%)
Age, mean ± SD, years	43.6 ± 9.9(20-99)	40.2	43.4	44.3
BMI, mean ± SD, kg/m²	23.4 ± 3.9	17.42	20.98	26.5
Bone Mass Density				
BMD, mean ± SD, g/cm²	1.13 ± 0.17	1.07	1.12	1.16
T-score, mean ± SD	-0.33 ± 1.24	-0.9	-0.52	-0.14
Z-score, mean ± SD	0.44 ± 1.13	-0.17	0.28	0.65
Suspected abnormal BMD (saBMD), n (%)	255 (11%)	33 (22.4%)	146 (13.5%)	69 (6.2%)
Non-saBMD, n (%)	2129 (89%)	114 (77.6%)	938 (86.5%)	1035 (93.8%)
Collected images				
Evaluable CXRs, n (%)	2335 (98%)	147 (100.0%)	1084 (99.1%)	1104 (96.6%)
Not Evaluable CXRs, n (%)	49 (2%)	0 (0.0%)	10 (0.9%)	39 (3.4%)

Data are presented as n (%) for categorical variables and mean ± standard deviation (SD) for continuous variables. saBMD = suspected abnormal bone mineral density (defined as T-score ≤ –2.5 for participants aged ≥ 50 years, or Z-score ≤ –2.0 for participants aged < 50 years); BMD = bone mineral density; CXR = chest X-ray. P-values were calculated using Student’s t-test for continuous variables and Chi-square test for categorical variables. Evaluable CXR refers to images meeting predefined

Variable	Overall (n = 2384)	BMI <18.5 N=147	BMI 18.5–23 N=1094	BMI ≥23 N=1143
Sex				

quality-control criteria (adequate thoracolumbar visualization, absence of artifacts or implants), whereas Not Evaluable CXR indicates images excluded for insufficient quality. These descriptive findings illustrate significant sex differences in BMI distribution, saBMD prevalence, and the proportion of evaluable chest radiographs, highlighting the importance of subgroup analyses in evaluating AI model performance and health equity.

Supplementary Table 4. Baseline characteristics by subgroup (sex × age × BMI category)

Age group	<50						≥50					
Sex	Female			Male			Female			Male		
BMI group	<18.5	18.5-23	≥23	<18.5	18.5-23	≥23	<18.5	18.5-23	≥23	<18.5	18.5-23	≥23
n	99	576	286	19	234	508	24	217	174	5	67	175
Age Mean±SD	36.5±7.3	39.4±6.7	39.9±6.2	36.2±8.7	38.1±7.2	39.0±6.4	54.4±2.8	55.9±6.0	56.7±6.0	60.7±15.5	56.3±7.1	54.3±3.0
BMI Mean±SD	17.48±0.79	20.76±1.21	26.39±3.31	17.50±0.80	21.43±1.10	26.88±3.14	17.22±1.72	20.92±1.20	25.99±3.05	16.80±0.86	21.45±1.06	26.09±2.46
BMD Mean±SD	1.10±0.11	1.17±0.13	1.22±0.14	1.07±0.16	1.11±0.12	1.17±0.13	0.94±0.16	1.00±0.16	1.06±0.14	1.04±0.13	1.06±0.14	1.14±0.15

T-score Mean±SD	-0.62±0.92	-0.11±1.05	0.33±1.13	-0.93±1.30	-0.57±1.01	-0.06±1.08	-1.95±1.30	-1.43±1.29	-0.96±1.18	-1.16±1.12	-0.97±1.12	-0.32±1.23
Z-score Mean±SD	-0.05±0.91	0.49±1.04	0.93±1.13	-0.43±1.29	-0.03±0.98	0.49±1.06	-0.48±1.23	0.15±1.12	0.69±1.02	-0.04±1.32	0.01±1.08	0.62±1.22
saBMD, n (%)	15 (15.2%)	36 (6.2%)	9 (3.1%)	6 (31.6%)	33 (14.3%)	21 (4.4%)	10 (41.7%)	67 (31.3%)	31 (18.1%)	2 (40.0%)	10 (15.6%)	8 (4.7%)
Non saBMD, n (%)	84 (84.8%)	540 (93.8%)	277 (96.9%)	13 (68.4%)	197 (85.7%)	457 (95.6%)	14 (58.3%)	147 (68.7%)	140 (81.9%)	3 (60.0%)	54 (84.4%)	161 (95.3%)
Evaluable, n (%)	99 (100.0%)	576 (100.0%)	286 (100.0%)	19 (100.0%)	230 (98.3%)	478 (94.1%)	24 (100.0%)	214 (98.6%)	171 (98.3%)	5 (100.0%)	64 (95.5%)	169 (96.6%)
Not Evaluable, n (%)	0 (0.0%)	0 (0.0%)	0 (0.0%)	0 (0.0%)	4 (1.7%)	30 (5.9%)	0 (0.0%)	3 (1.4%)	3 (1.7%)	0 (0.0%)	3 (4.5%)	6 (3.4%)

Data are presented as n (%) for categorical variables and mean ± standard deviation (SD) for continuous variables. saBMD = suspected abnormal bone mineral density (defined as T-score ≤ –2.5 for participants aged ≥ 50 years, or Z-score ≤ –2.0 for participants aged < 50 years); BMD = bone mineral density; CXR = chest X-ray. P-values were calculated using Student's t-test for continuous variables and Chi-square test for categorical variables. Evaluable CXR refers to images meeting predefined quality-control criteria (adequate thoracolumbar visualization, absence of artifacts or implants), whereas Not Evaluable CXR indicates images excluded for insufficient quality. These descriptive findings illustrate significant sex differences in BMI distribution, saBMD prevalence, and the proportion of evaluable chest radiographs, highlighting the importance of subgroup analyses in evaluating AI model performance and health equity.

Supplementary Table 5. Comparison of BMI category and sex-stratified distributions across our study cohort, Taiwanese NAHSIT, Japanese NHNS, and Korean KNHANES data.

BMI Category / Sex	Our Study Cohort (%)	Taiwan NAHSIT (2017–2020)	Japan NHNS (2022) (%)	Korea KNHANES (2021–2023) (%)	Notes

		(%)			
Underweight (BMI <18.5), Male	2.4	4.1	4.4	2.3	
Underweight (BMI <18.5), Female	8.9	10.6	11.0	13.7 (age 20–39)	KNHANES age-stratified
Normal weight (18.5–23), Male	29.2	43.5	36.7	36.0	
Normal weight (18.5–23), Female	57.4	43.5	45.8	51.4	
Overweight (BMI ≥23), Male	68.5	47.9	58.9	61.7	
Overweight (BMI ≥23), Female	30.1	47.9	43.2	34.9	
Mean BMI, Male	24.9	24.0	23.7	24.6	
Mean BMI, Female	22.0	23.0	21.8	23.1	

Supplementary Table 6. Subgroup-specific diagnostic performance and prevalence-standardized predictive values of the AI model across sex, age, and WHO BMI categories

(a) Diagnostic performance by observed prevalence (St. Paul’s cohort)

**Subgroup**	**N**	**Disease prevalence %**	**Sensitivity %**	**Specificity %**	**PPV %**	**NPV %**	**LR+**	**LR−**	**AUC (DeLong)**

Female <50, BMI <18.5	99	1.01 (0.03–5.50)	100.00 (2.50–100.00)	85.71 (77.19–91.96)	6.67 (4.21–10.40)	100.00 (95.70–100.00)	7.00 (4.31–11.37)	0.00	0.949 (0.898–0.990)
Female <50, BMI 18.5–23	576	0.69 (0.19–1.77)	75.00 (19.41–99.37)	94.23 (91.99–96.00)	8.33 (4.51–14.90)	99.81 (99.00–99.97)	13.00 (6.75–25.04)	0.27 (0.05–1.45)	0.980 (0.953–1.000)
Female <50, BMI ≥23	286	0.35 (0.01–1.93)	100.00 (2.50–100.00)	97.19 (94.54–98.78)	11.11 (5.94–19.84)	100.00 (98.68–100.00)	35.63 (17.99–70.54)	0.00	0.990 (0.975–1.000)
Male <50, BMI <18.5	19	15.79 (3.38–39.58)	100.00 (29.24–100.00)	81.25 (54.35–95.95)	50.00 (26.50–73.50)	100.00 (75.29–100.00)	5.33 (1.92–14.79)	0.00	0.938 (0.824–1.000)
Male <50, BMI 18.5–23	230	2.61 (0.96–5.59)	100.00 (54.07–100.00)	87.95 (82.95–91.90)	18.18 (13.50–24.04)	100.00 (98.14–100.00)	8.30 (5.82–11.82)	0.00	0.992 (0.979–1.000)
Male <50, BMI ≥23	478	0.84 (0.23–2.13)	100.00 (39.76–100.00)	96.41 (94.32–97.90)	19.05 (12.86–27.29)	100.00 (99.20–100.00)	27.88 (17.48–44.47)	0.00	0.981 (0.963–0.998)
Female ≥50, BMI <18.5	24	33.33 (15.63–55.32)	100.00 (63.06–100.00)	87.50 (61.65–98.45)	80.00 (52.25–93.60)	100.00 (76.84–100.00)	8.00 (2.19–29.25)	0.00	0.953 (0.876–1.000)
Female ≥50, BMI 18.5–23	214	21.96 (16.61–28.11)	89.36 (76.90–96.45)	85.03 (78.70–90.07)	62.69 (53.60–70.96)	96.60 (92.52–98.49)	5.97 (4.10–8.68)	0.13 (0.05–0.29)	0.929 (0.891–0.968)
Female ≥50, BMI	171	10.53 (6.36–16.1	94.44 (72.71–99.86)	90.85 (85.12–94.91	54.84 (42.13–66.95	99.29 (95.39–99.89)	10.32 (6.19–17.22)	0.06 (0.01–0.41	0.967 (0.938–0.995

≥23		3)		)	)			)	)
Male ≥50, BMI <18.5	5	20.00 (0.51–71.64)	100.00 (2.50–100.00)	75.00 (19.41–99.37)	50.00 (15.48–84.52)	100.00 (29.24–100.00)	4.00 (0.73–21.84)	0.00	1.000 (1.000–1.000)
Male ≥50, BMI 18.5–23	64	9.38 (3.52–19.30)	83.33 (35.88–99.58)	91.38 (81.02–97.14)	50.00 (28.68–71.32)	98.15 (89.84–99.69)	9.67 (3.89–24.04)	0.18 (0.03–1.09)	0.960 (0.903–1.000)
Male ≥50, BMI ≥23	169	1.78 (0.37–5.10)	100.00 (29.24–100.00)	96.99 (93.11–99.01)	37.50 (20.20–58.72)	100.00 (97.73–100.00)	33.20 (14.00–78.71)	0.00	0.986 (0.966–1.000)

(b) **Standardized predictive values at plausible prevalence levels (men 7%; women 10% and 25%) and workload/yield indices**

Standardization was performed using Bayes' theorem at plausible prevalence levels derived from East Asian community data (men 7%; women 10% and 25%). Each female subgroup is presented at both 10% and 25% prevalence to illustrate the effect of population prevalence on predictive values and efficiency indices.

Subgroup	Prevalence used (%)	PPV_std %	PPV_std 95% CI	NPV_std %	NPV_std 95% CI	Positives per 100 screened	Cases per 100 AI⁺	DXA per detected case	Cases per 100 screened	NNS
Female <50, BMI <18.5	10	40.00	(28.00–55.00)	98.50	(95.00–100.00)	35.71	40.00	2.50	14.29	7.00
Female <50, BMI <18.5	25	70.00	(60.87–80.33)	100.00	(100.00–100.00)	35.71	70.00	1.43	25.00	4.00
Female <50, BMI 18.5–23	10	39.90	(31.50–49.50)	98.60	(97.20–99.50)	23.08	39.90	2.50	9.19	10.9

Subgroup	Prevalence used (%)	PPV_std %	PPV_std 95% CI	NPV_std %	NPV_std 95% CI	Positives per 100 screened	Cases per 100 AI⁺	DXA per detected case	Cases per 100 screened	NNS
Female <50, BMI 18.5–23	25	81.25	(59.09–88.41)	91.88	(79.03–100.00)	23.08	81.25	1.23	18.75	5.33
Female <50, BMI ≥23	10	53.40	(40.80–66.00)	99.30	(97.20–99.90)	27.11	53.40	1.87	14.47	6.91
Female <50, BMI ≥23	25	92.23	(87.16–96.94)	100.00	(100.00–100.00)	27.11	92.23	1.08	25.00	4.00
Male <50, BMI <18.5	7	28.64	(16.72–100.00)	100.00	(100.00–100.00)	24.44	28.64	3.49	7.00	14.29
Male <50, BMI 18.5–23	7	38.44	(31.30–48.37)	100.00	(100.00–100.00)	18.21	38.44	2.60	7.00	14.29
Male <50, BMI ≥23	7	67.73	(58.80–79.86)	100.00	(100.00–100.00)	10.34	67.73	1.48	7.00	14.29
Female ≥50, BMI <18.5	10	47.10	(22.90–79.60)	100.00	(96.60–100.00)	34.38	47.10	2.12	16.19	6.18
Female ≥50, BMI <18.5	25	72.73	(51.61–100.00)	100.00	(100.00–100.00)	34.38	72.73	1.38	25.00	4.00
Female ≥50, BMI 18.5–23	10	39.90	(31.50–49.50)	98.60	(97.20–99.50)	33.57	39.90	2.50	13.43	7.45
Female ≥50,	25	66.55	(58.70–75.40)	96.00	(92.54–99.16)	33.57	66.55	1.50	22.34	4.48

Subgroup	Prevalence used (%)	PPV_std %	PPV_std 95% CI	NPV_std %	NPV_std 95% CI	Positives per 100 screened	Cases per 100 AI⁺	DXA per detected case	Cases per 100 screened	NNS
BMI 18.5–23										
Female ≥50, BMI ≥23	10	53.40	(40.80–66.00)	99.30	(97.20–99.90)	30.47	53.40	1.87	16.21	6.17
Female ≥50, BMI ≥23	25	77.48	(68.65–86.44)	98.00	(94.04–100.00)	30.47	77.48	1.29	23.61	4.24
Male ≥50, BMI <18.5	7	23.14	(9.12–100.00)	100.00	(100.00–100.00)	30.25	23.14	4.32	7.00	14.29
Male ≥50, BMI 18.5–23	7	42.12	(24.44–78.44)	98.65	(95.97–100.00)	13.85	42.12	2.37	5.83	17.14
Male ≥50, BMI ≥23	7	71.42	(55.54–92.59)	100.00	(100.00–100.00)	9.80	71.42	1.40	7.00	14.29

Notes. Sensitivity = TP/(TP+FN); Specificity = TN/(TN+FP); PPV = TP/(TP+FP); NPV = TN/(TN+FN); LR+ = Sensitivity/(1–Specificity); LR– = (1–Sensitivity)/Specificity. 95% CIs: exact (Clopper–Pearson) or Wilson for proportions (exact for small denominators); AUC 95% CIs by DeLong; if DeLong variance is 0/unstable, stratified bootstrap CIs are reported. LRs use log-transformed approximations. Standardized PPV/NPV at 5% prevalence derived via Bayes' theorem for cross-subgroup comparability. For participants <50 years, results are labeled "low BMD for age" per ISCD; considered secondary analyses. Efficiency metrics: Positives per 100 screened = AI-positive per 100 screened; Cases per 100 AI+ = PPV×100; DXA per detected case = 1/PPV; Cases per 100 screened = (Positives per 100)×(PPV/100); NNS = 100/(Cases per 100 screened). Abbreviations: TP, true positive; FP, false positive; TN, true negative; FN, false negative; AUC, area under ROC; BMI, body mass index; WHO, World Health Organization; ISCD, International Society for Clinical Densitometry.

Supplementary Dataset 1. Characteristics of AI-Detected True Positive Cases

Summary of AI-detected true positive cases based on DXA T-scores (for participants aged ≥50 years) or Z-scores (for those aged <50 years).

No.	(Age, sex, BMI)	BMI	Age	sex	DXA BMD	DXA T-score	DXA Z-score
1	<50, F, <18.5	16.8	41.9	Female	0.846	-2.7	-2.2
2	<50, F, 18.5-23	21	41.3	Female	0.836	-2.8	-2.3
3	<50, F, 18.5-23	21.1	43.5	Female	0.865	-2.6	-2
4	<50, F, 18.5-23	19.9	48.1	Female	0.747	-3.6	-2.7
5	<50, F, ≥23	23.8	38.9	Female	0.854	-2.7	-2.1
6	<50, M, <18.5	18	49.3	Male	0.829	-2.9	-2.1
7	<50, M, <18.5	17.4	33.7	Male	0.873	-2.5	-2.1
8	<50, M, <18.5	17.7	32.9	Male	0.839	-2.8	-2.4
9	<50, M, 18.5-23	18.9	48	Male	0.780	-3.3	-2.5
10	<50, M, 18.5-23	22	36.3	Male	0.820	-3	-2.5
11	<50, M, 18.5-23	21.5	45.9	Male	0.808	-3.1	-2.3
12	<50, M, 18.5-23	21.2	40.2	Male	0.868	-2.6	-2
13	<50, M, 18.5-23	21.4	40.8	Male	0.714	-3.8	-3.2
14	<50, M, 18.5-23	21.1	39.9	Male	0.861	-2.6	-2
15	<50, M, ≥23	25.4	43.1	Male	0.839	-2.8	-2.1

16	<50, M, ≥23	24.8	39.2	Male	0.781	-3.3	-2.7
17	<50, M, ≥23	24.9	39.7	Male	0.857	-2.6	-2.1
18	<50, M, ≥23	23.4	42.5	Male	0.840	-2.8	-2.2
19	≥50, F, <18.5	17.3	56.8	Female	0.655	-4.3	-2.6
20	≥50, F, <18.5	18.1	55.1	Female	0.800	-3.1	-1.5
21	≥50, F, <18.5	14.9	56.7	Female	0.750	-3.5	-1.8
22	≥50, F, <18.5	18.4	53.2	Female	0.768	-3.4	-2
23	≥50, F, <18.5	17.3	52.6	Female	0.846	-2.7	-1.4
24	≥50, F, <18.5	15.8	54.1	Female	0.635	-4.5	-3
25	≥50, F, <18.5	10.4	60.2	Female	0.803	-3.1	-1
26	≥50, F, <18.5	18	54.2	Female	0.727	-3.7	-2.2
27	≥50, F, 18.5-23	21.8	58.7	Female	0.745	-3.6	-1.6
28	≥50, F, 18.5-23	21.3	57.7	Female	0.872	-2.5	-0.7
29	≥50, F, 18.5-23	21.4	59.9	Female	0.699	-4	-1.9
30	≥50, F, 18.5-23	22.2	59.3	Female	0.877	-2.5	-0.5
31	≥50, F, 18.5-23	20.8	58.7	Female	0.780	-3.3	-1.3
32	≥50, F, 18.5-23	21.4	58.4	Female	0.815	-3	-1.1
33	≥50, F, 18.5-23	18.8	53.4	Female	0.877	-2.5	-1.1
34	≥50, F, 18.5-23	19.5	73.1	Female	0.748	-3.6	-0.5

35	≥50, F, 18.5-23	22.9	58.2	Female	0.757	-3.5	-1.6
36	≥50, F, 18.5-23	21.1	57.8	Female	0.760	-3.5	-1.6
37	≥50, F, 18.5-23	20.6	55.9	Female	0.838	-2.8	-1.1
38	≥50, F, 18.5-23	20.3	51.3	Female	0.801	-3.1	-1.9
39	≥50, F, 18.5-23	19.9	51	Female	0.859	-2.6	-1.5
40	≥50, F, 18.5-23	19.6	56	Female	0.763	-3.4	-1.8
41	≥50, F, 18.5-23	19.7	54.7	Female	0.859	-2.6	-1.1
42	≥50, F, 18.5-23	22.7	56.3	Female	0.838	-2.8	-1.1
43	≥50, F, 18.5-23	19.1	59.1	Female	0.775	-3.3	-1.3
44	≥50, F, 18.5-23	20.4	71.2	Female	0.738	-3.6	-0.7
45	≥50, F, 18.5-23	21.2	50.3	Female	0.842	-2.8	-1.7
46	≥50, F, 18.5-23	21.6	52.9	Female	0.857	-2.6	-1.3
47	≥50, F, 18.5-23	21.4	52.8	Female	0.84	-2.8	-1.4
48	≥50, F, 18.5-23	18.9	54	Female	0.746	-3.6	-2.1
49	≥50, F, 18.5-23	21.4	57.5	Female	0.840	-2.8	-1
50	≥50, F, 18.5-23	22.2	54.4	Female	0.869	-2.5	-1
51	≥50, F, 18.5-23	19.7	58.3	Female	0.777	-3.3	-1.4
52	≥50, F, 18.5-23	22.3	55.7	Female	0.795	-3.2	-1.5
53	≥50, F, 18.5-23	20.9	69.3	Female	0.726	-3.7	-0.9

54	≥50, F, 18.5-23	19.7	60.6	Female	0.824	-2.9	-0.8
55	≥50, F, 18.5-23	22.7	59.3	Female	0.802	-3.1	-1.1
56	≥50, F, 18.5-23	21.2	51.9	Female	0.800	-3.1	-1.9
57	≥50, F, 18.5-23	19.3	58.4	Female	0.826	-2.9	-1
58	≥50, F, 18.5-23	21.2	55.1	Female	0.764	-3.4	-1.8
59	≥50, F, 18.5-23	19.6	54.5	Female	0.796	-3.2	-1.6
60	≥50, F, 18.5-23	19.3	57.6	Female	0.737	-3.6	-1.8
61	≥50, F, 18.5-23	22.8	81.8	Female	0.813	-3	0.1
62	≥50, F, 18.5-23	18.7	76.2	Female	0.649	-4.4	-1.3
63	≥50, F, 18.5-23	21.8	54.6	Female	0.766	-3.4	-1.9
64	≥50, F, 18.5-23	22.9	78.8	Female	0.826	-2.9	0.1
65	≥50, F, 18.5-23	22.5	63.8	Female	0.776	-3.3	-0.8
66	≥50, F, 18.5-23	20.1	58.4	Female	0.835	-2.8	-0.9
67	≥50, F, 18.5-23	19.5	81.8	Female	0.680	-4.1	-1
68	≥50, F, 18.5-23	22.1	55.4	Female	0.805	-3.1	-1.5
69	≥50, F, ≥23	23.2	65	Female	0.855	-2.7	-0.1
70	≥50, F, ≥23	25.4	66.2	Female	0.842	-2.8	-0.1
71	≥50, F, ≥23	23.6	59.1	Female	0.759	-3.5	-1.5
72	≥50, F, ≥23	23.2	58.8	Female	0.880	-2.5	-0.5

73	≥50, F, ≥23	27.6	59.2	Female	0.819	-3	-1
74	≥50, F, ≥23	23.8	57	Female	0.843	-2.8	-1
75	≥50, F, ≥23	23.8	56.9	Female	0.773	-3.3	-1.6
76	≥50, F, ≥23	23.5	54.2	Female	0.870	-2.5	-1
77	≥50, F, ≥23	23.5	65.2	Female	0.877	-2.5	0
78	≥50, F, ≥23	26.1	61.4	Female	0.809	-3	-0.8
79	≥50, F, ≥23	25.7	53.8	Female	0.867	-2.6	-1.1
80	≥50, F, ≥23	25.9	61.1	Female	0.759	-3.5	-1.3
81	≥50, F, ≥23	24	74.1	Female	0.873	-2.5	0.5
82	≥50, F, ≥23	28.8	63.4	Female	0.853	-2.7	-0.2
83	≥50, F, ≥23	24	58.8	Female	0.838	-2.8	-0.9
84	≥50, F, ≥23	23.6	85.9	Female	0.817	-3	0.1
85	≥50, F, ≥23	24.1	68.3	Female	0.775	-3.3	-0.6
86	≥50, M, <18.5	16.6	53.7	Male	0.839	-2.8	-1.8
87	≥50, M, 18.5-23	22.5	94.2	Male	0.858	-2.6	-0.6
88	≥50, M, 18.5-23	19.2	52.7	Male	0.830	-2.9	-2
89	≥50, M, 18.5-23	21.1	56	Male	0.740	-3.6	-2.6
90	≥50, M, 18.5-23	21.8	58.9	Male	0.818	-3	-1.9
91	≥50, M, 18.5-23	19.4	50.7	Male	0.827	-2.9	-2

92	≥50, M, ≥23	24.4	53.1	Male	0.850	-2.7	-1.8
93	≥50, M, ≥23	23	56.5	Male	0.777	-3.3	-2.3
94	≥50, M, ≥23	23.3	53.2	Male	0.826	-2.9	-2

Supplementary Table 7. Exploratory mapping of AI-identified true positives (n=94) against guideline-based eligibility (proxy definitions)

Guideline	Category	n (%)	Notes / Caveats
ISCD 2023 (proxy: age, sex, BMI only*)	Eligible	27 (28.7)	Proxy definition excludes clinical risk factors (e.g., menopausal status, fracture history, medications); true eligibility likely underestimated
	Ineligible	67 (71.3)	Would not qualify under proxy-only criteria
USPSTF 2025	Women ≥65 years (screen)	13 (13.8)	Screening universally recommended
	Women <65 years (risk-based)	59 (62.8)	Requires additional risk factor assessment not available in dataset
	Men (evidence insufficient)	22 (23.4)	Screening not routinely recommended

Supplementary Table 8. Model card

Dataset	Training	Validation	Pre-clinical testing
Number	4188<br><br>(aged ≥50: 3731; aged <50: 457)	400<br><br>(aged ≥50: 334; aged <50: 66)	534<br><br>(aged ≥50: 435; aged <50: 99)
Age	20~99 years old	22~98 years old	26~96 years old
Image resolution	Rows: 1752~4280<br><br>Cols: 1656~4280	Rows: 1760~4280<br><br>Cols: 1760~3520	Rows: 1760~4280<br><br>Cols: 1760~4280
kVp	68~124	70~120	70~120
mAs	1~25	2~18	2~20
Positive cases	1129	108	153
Negative cases	3059	292	381

VeriOsteo OP utilizes a deep learning model based on the Vision Transformer (ViT-Large) architecture. During training, input chest X-ray images were resized to 448 × 448 pixels and partitioned into non-overlapping 16 × 16 patches, which were processed by a Transformer encoder (24 layers) followed by a multilayer perception (MLP) head to predict BMD. The model was trained on paired chest radiograph–DXA data, with DXA-derived L1–L4 BMD serving as the reference standard. Model outputs included both a continuous BMD estimate and a binary classification of suspected abnormal bone density, mapped to T- or Z-score thresholds based on age. The algorithm was locked after training, meaning that the model weights and decision thresholds were fixed and did not change over time.

The model's training dataset was retrospectively collected from hospitals in Taiwan. Image quality was reviewed by a senior orthopedic physician to confirm adequate coverage of the chest and sufficient exposure. A total of 4,188 chest X-ray images were used for training and 400 images for validation. Among these, 1,237 images (27%) were classified as suspected abnormal bone mineral density (saBMD) and 3,351 images

(73%) as non-saBMD. An additional 534 images were reserved for pre-clinical testing, yielding a sensitivity of 0.90, a specificity of 0.86, and an AUC of 0.94.

The BMD value of lumbar vertebrae L1-L4, measured by central dual-energy x-ray absorptiometry (DXA), served as the reference standard. These values were then converted to T-score or Z-score using the equation respectively below [1, 2].

$$T - score = \frac{(Measured\ BMD - mean\ BMD\ of\ the\ reference\ population)}{the\ reference\ population's\ standard\ deviation\ (SD)} \quad \text{(Equation 1)}$$

The T-score relies on a reference population. The formula employs the average BMD values of young Caucasian women (aged 20-29 from the Third National Health and Nutrition Examination Survey, NHANES III database) as the benchmark, a recommendation also endorsed by the International Society for Clinical Densitometry (ISCD) [3, 4].

$$Z - score = \frac{(Measured\ BMD - mean\ BMD\ of\ age\ matched\ population)}{age\ matched\ population's\ standard\ deviation\ (SD)} \quad \text{(Equation 2)}$$

The reference values for the Z-score are calculated based on the mean and standard deviation of bone mineral density within the same ethnicity, gender, and age group.

Among the values calculated from lumbar vertebrae L1-L4, the lowest T-score or Z-score is selected. If the T-score is ≤ -2.5 or the Z-score is ≤ -2.0, it is classified as a positive case indicating abnormal bone density. Conversely, if the T-score or Z-score is above these thresholds, it is classified as a negative case indicating no significant abnormality in bone density.

References:

[1] Cosman, F, et al. National Osteoporosis Foundation. Clinician's Guide to Prevention and Treatment of Osteoporosis. *Osteoporos Int.* Oct 2014.

[2] Blake, Glen M and Fogelman, Ignac. The role of DXA bone density scans in the diagnosis and treatment of osteoporosis. *Postgrad Med J.* Aug 2007.

[3] Hwang, Jawl-Shan, Chen, Jung-Fu and Tsai, Keh-Sung. Epidemiology of Osteoporosis in Taiwan. *Osteoporosis of the Spine.* 2021.

[4] 2019 ISCD Official Positions Adult. The International Society for Clinical Densitometry. [Online] 2019. https://iscd.org/learn/official-positions/adult-positions/.

Supplementary Table 9. DXA Reference Database (NHANES III and St. Paul's Hospital Device Parameters)

Parameter	Specification / Reference	Notes
**DXA system**	GE Lunar Prodigy Advance (GE Healthcare, Madison, WI, USA)	Installed at St. Paul's Hospital Health Examination Center
**Software version**	enCORE Version 18 (GE Healthcare)	Consistent with NHANES III reference settings
**Scan region**	Lumbar spine (L1–L4)	Used for all analyses
**Evaluation criteria**	All four vertebrae included; T-score difference ≤ 1 between adjacent vertebrae	Excluded scans not meeting these criteria
**Phantom calibration**	Daily quality control using GE Lunar phantom	Conducted per manufacturer protocol
**Precision (Coefficient of Variation, CV)**	< 1.5% for lumbar spine BMD	Based on repeat phantom and patient measurements at St. Paul's Hospital
**Reference database for T-score**	Young Caucasian women aged 20–29 years from NHANES III (Third National Health and Nutrition Examination Survey)	Recommended by WHO and ISCD Official Positions (2019)
**Reference database for Z-score**	Age-, sex-, and ethnicity-matched reference values from the GE Lunar Asian database	Used for participants < 50 years old ("low BMD for age")
**Output variables**	BMD (g/cm^2), T-score, Z-score	Lowest vertebra (L1–L4) value used as final result
**Quality control summary**	Daily phantom stability confirmed throughout study period (2012–2023)	No drift exceeding manufacturer tolerance (±1%)

Abbreviations: BMD, bone mineral density; CV, coefficient of variation; DXA, dual-energy X-ray absorptiometry; ISCD, International Society for Clinical Densitometry; NHANES, National Health and Nutrition Examination Survey; WHO, World Health Organization.

Supplementary Figure 1. DXA System and Reference Data (GE Lunar enCORE outputs)

Representative screenshots and tabulated outputs from the GE Lunar Prodigy Advance DXA system (enCORE Version 18) are shown, including:

1. System configuration and phantom calibration log demonstrating daily quality control stability.
2. Reference curve and standard deviation parameters used for T-score conversion based on NHANES III young adult female data.
3. Example lumbar spine (L1–L4) BMD report illustrating automated output of BMD (g/cm²), T-score, and Z-score values.

These materials document the reference database and calibration consistency of the St. Paul's Hospital DXA system used in the present study.

Female Spine L1-L4

	YA	SD	25 yr	35 yr	45 yr	55 yr	65 yr	75 yr	85 yr
Asia	1.112	0.12	1.112	1.112	1.112	0.992	0.872	0.807	0.797
Australia/Geelong	1.207	0.1324	1.207	1.207	1.207	1.135	1.063	0.994	0.925
USA/N. Europe	1.180	0.12	1.180	1.180	1.180	1.085	0.990	0.970	0.950
China	1.114	0.12	1.114	1.114	1.114	0.991	0.868	0.792	0.762
Egypt	1.150	0.12	1.150	1.150	1.150	1.050	0.950	0.940	0.930
Finland	1.170	0.12	1.170	1.170	1.170	1.045	0.920	0.900	0.880
France	1.160	0.12	1.160	1.160	1.105	0.995	0.885	0.855	0.825
Indonesia	1.110	0.12	1.110	1.110	1.110	1.025	0.940	0.878	0.816
Italy	1.180	0.12	1.180	1.180	1.130	1.030	0.930	0.900	0.870
Japan	1.110	0.12	1.110	1.110	1.110	0.990	0.870	0.840	0.810
Korea	1.149	0.12	1.149	1.149	1.149	1.051	0.953	0.843	0.733
Mexico	1.170	0.12	1.170	1.170	1.120	1.020	0.920	0.895	0.870
Middle East	1.101	0.12	1.101	1.101	1.101	1.021	0.941	0.931	0.921
Philippines	1.110	0.12	1.110	1.110	1.110	0.988	0.866	0.835	0.804
Spain	1.180	0.12	1.180	1.180	1.130	1.030	0.930	0.900	0.870
Tunisia	1.148	0.12	1.148	1.148	1.148	1.018	0.888	0.858	0.828
Turkey	1.136	0.12	1.136	1.136	1.136	1.056	0.976	0.906	0.836

Male Spine L1-L4

	YA	SD	25 yr	35 yr	45 yr	55 yr	65 yr	75 yr	85 yr
Asia	1.131	0.12	1.139	1.124	1.098	1.062	1.026	0.993	0.963
USA/N. Europe	1.220	0.12	1.220	1.220	1.210	1.189	1.168	1.147	1.126
China	1.082	0.12	1.095	1.070	1.045	1.022	0.999	0.980	0.966
Finland	1.210	0.12	1.210	1.210	1.200	1.180	1.160	1.140	1.120
France	1.190	0.12	1.190	1.190	1.179	1.158	1.137	1.116	1.095
Indonesia	1.095	0.12	1.095	1.095	1.095	1.090	1.085	1.075	1.065
Italy	1.220	0.12	1.220	1.220	1.210	1.189	1.168	1.147	1.126
Japan	1.180	0.12	1.180	1.180	1.155	1.105	1.055	1.005	0.955
Korea	1.180	0.12	1.180	1.180	1.155	1.105	1.055	1.005	0.955
Mexico	1.180	0.12	1.180	1.180	1.165	1.135	1.105	1.075	1.045
Middle East	1.112	0.12	1.112	1.112	1.112	1.082	1.052	1.042	1.032
Spain	1.220	0.12	1.220	1.220	1.210	1.189	1.168	1.147	1.126
Turkey	1.112	0.12	1.112	1.112	1.112	1.082	1.052	1.042	1.032

Supplementary Figure 2. Standardized predictive values and prevalence-dependent performance of the AI model in adults ≥ 50 years. Panels show the relationship between positive predictive value (PPV, bottom) and negative predictive value (NPV, top) across plausible prevalence levels (x-axis) for women (left) and men (right) aged ≥ 50 years, stratified by WHO Asia–Pacific BMI categories (< 18.5, 18.5–23, ≥ 23 kg/m²). Curves were derived using Bayes' theorem from subgroup-specific likelihood ratios (LR^+, LR^-) obtained in the validation dataset (Supplementary Table S6). At low disease prevalence, NPV remained high across all strata (96–100%), indicating strong rule-out reliability, whereas PPV increased proportionally with prevalence and BMI, reaching ≈40–54% at 10% and ≈67–77% at 25% for women, and ≈42–71% at 7% for men. These findings illustrate the model's capacity to reduce unnecessary DXA referrals while prioritizing individuals most likely to benefit from confirmatory testing.

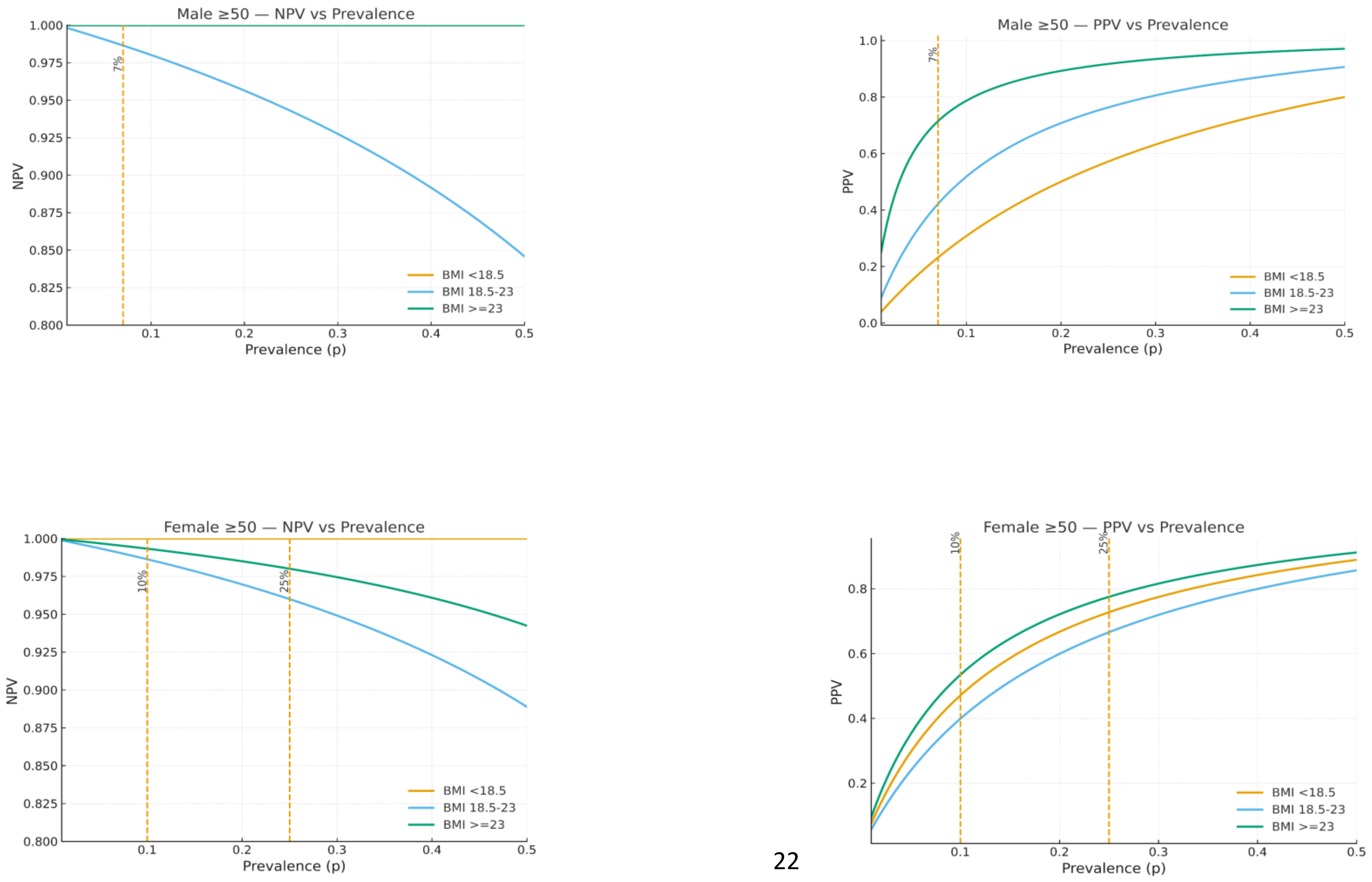

Supplement: Supplementary file 1 — Supplementary Information [file 41746_2026_2484_MOESM1_ESM.pdf]
